# A NAC transcription factor, NOR-like1, is a new positive regulator of tomato fruit ripening

**DOI:** 10.1038/s41438-018-0111-5

**Published:** 2018-12-21

**Authors:** Ying Gao, Wei Wei, Xiaodan Zhao, Xiaoli Tan, Zhongqi Fan, Yiping Zhang, Yuan Jing, Lanhuan Meng, Benzhong Zhu, Hongliang Zhu, Jianye Chen, Cai-Zhong Jiang, Donald Grierson, Yunbo Luo, Da-Qi Fu

**Affiliations:** 1 Laboratory of Fruit Biology, College of Food Science & Nutritional Engineering, China Agricultural University, 100083 Beijing, China; 2 College of Horticulture, South China Agricultural University, 510642 Guangzhou, China; 3 College of Food Science, Beijing Technology and Business University, 100037 Beijing, China; 4 Department of Plant Sciences, University of California, Davis, CA 95616 USA; 5 Crops Pathology and Genetics Research Unit, United States Department of Agriculture, Agricultural Research Service, Davis, CA 95616 USA; 6 Plant Sciences Division, School of Biosciences, University of Nottingham, Sutton Bonington Campus, Loughborough, LE12 5RD UK; 7 College of Agriculture & Biotechnology, Zhejiang University, 310058 Hangzhou, China

## Abstract

Ripening of the model fruit tomato (*Solanum lycopersicum*) is controlled by a transcription factor network including NAC (NAM, ATAF1/2, and CUC2) domain proteins such as No-ripening (NOR), SlNAC1, and SlNAC4, but very little is known about the NAC targets or how they regulate ripening. Here, we conducted a systematic search of fruit-expressed NAC genes and showed that silencing *NOR-like1* (Solyc07g063420) using virus-induced gene silencing (VIGS) inhibited specific aspects of ripening. Ripening initiation was delayed by 14 days when NOR-like1 function was inactivated by CRISPR/Cas9 and fruits showed obviously reduced ethylene production, retarded softening and chlorophyll loss, and reduced lycopene accumulation. RNA-sequencing profiling and gene promoter analysis suggested that genes involved in ethylene biosynthesis (*SlACS2*, *SlACS4*), color formation (*SlGgpps2*, *SlSGR1*), and cell wall metabolism (*SlPG2a*, *SlPL*, *SlCEL2,* and *SlEXP1*) are direct targets of NOR-like1. Electrophoretic mobility shift assays (EMSA), chromatin immunoprecipitation-quantitative PCR (ChIP-qPCR), and dual-luciferase reporter assay (DLR) confirmed that NOR-like1 bound to the promoters of these genes both in vitro and in vivo, and activated their expression. Our findings demonstrate that NOR-like1 is a new positive regulator of tomato fruit ripening, with an important role in the transcriptional regulatory network.

## Crop genetics: novel gene involved in tomato ripening

Chinese researchers have identified a new gene which regulates the ripening of tomatoes. Several genes known to control tomato ripening are members of the NAC family of regulators. To identify others, a team led by Daqi Fu of China Agricultural University blocked the expression of candidate NAC genes. The discovered that silencing *NOR-like1* repressed ripening, leaving the tomatoes partially green. The team also engineered plants with defective copies of *NOR-like1* and found that this delayed ripening and eventually resulted in partially ripe fruit and impaired seed development. RNA sequencing of these lines revealed that *NOR-like1* directly regulates genes involved in ethylene synthesis, carotenoid accumluation, chlorophyll metabolism, and cell wall breakdown. These findings clearly demonstrate a key role for *NOR-like1* as a positive regulator of tomato ripening and a potential tool for controlling this important process.

## Introduction

Tomato fruit ripening is regulated by endogenous hormones, environmental signals and genetic regulators acting in a network that determines the specific expression of ripening-associated genes^1–3^. Transcriptional regulation has a very important role during tomato fruit ripening and the identification of target genes of transcription factors (TFs) is important for understanding the mechanism of ripening regulation^4,5^.

Early studies on the transcriptional regulation of fruit ripening were based on the characterization of natural fruit ripening mutants^6^ such as ripening-inhibitor (*rin*)^7^ and colorless non-ripening (*Cnr*)^8^. MADS-RIN was identified by the cloning of the *rin* mutant, which inhibited almost all measured ripening phenomena and the mutation was caused by a deletion of a genomic DNA fragment between *RIN* and *macrocalyx* (*MC) *, forming a chimeric gene (*RIN-MC*) that was thought to be a loss of function mutation. MADS-RIN was considered to regulate ripening, whereas MADS-MC was thought to affect sepal development and inflorescence determinacy^7^. However, the role of MADS-RIN in tomato fruit ripening was recently re-assessed in studies that showed the mutated *RIN-MC* was actually translated into a functional transcription factor to regulate many genes involved in tomato fruit ripening^9,10^. The *Cnr* mutant, which is colorless and has altered fruit pericarp texture, results from a spontaneous epigenetic change in the SBP-box gene *LeSPL-CNR* promoter, whereas the *CNR* gene sequence, itself remains unaltered^8^. Map-based cloning showed the *no-ripening* (*nor)* mutant was due to the loss of two A bases in the *NOR* coding sequence, which resulted in the early termination of protein translation (Patent No.: US 6,762,347 B1). Yuan et al.^11^ compared the proteome of the *nor* mutant with that in wild-type fruits used isobaric tags for relative and absolute quantitation (iTRAQ) and showed that many proteins involved in fruit ripening, quality and disease resistance were altered. NOR is a NAC family transcription factor, having a global role in tomato fruit ripening, but compared with the other two ripening-related mutants *rin* and *Cnr*, the mechanism NOR regulate tomato fruit ripenin*g* is unclear. A recent study confirmed that *NAC-NOR* mutations in tomato *Penjar* accessions attenuated multiple metabolic processes and prolonged fruit shelf life^12^.

Identification of other crucial ripening-associated TFs has recently shed more light on the complex mechanism of tomato fruit ripening. TAGL1, a MADS transcription factor, directly activated the expression of ethylene biosynthetic genes *SlACS2* in tomato fruits^13^. *TAGL1*-silenced fruits remain yellow and ethylene production is decreased significantly^14^. Two additional MADS family TFs, FUL1/FUL2 also have crucial roles in tomato fruit ripening. FUL1/FUL2-silenced tomato fruits are orange-ripe and do not regulate ethylene biosynthesis^15^. Fujisawa et al.^16^ demonstrated that FUL homologs, RIN, and TAGL1 form a DNA-binding complex, and probably regulate tomato fruit ripening through tetrameric complexes. Another MADS TF, SlMADS1, can interact with RIN, but acts as a negative regulator of fruit ripening^17^. SlAP2a is a negative regulator of ethylene biosynthesis, and *SlAP2a*-RNAi tomato lines over-produce ethylene^18^. An additional TF, LeHB-1, encodes a homeobox protein that binds in vitro to the promoter of the ethylene biosynthesis gene *LeACO1*, which encodes the final enzyme required for ethylene synthesis^19^.

NAC TFs (comprising NAM, ATAF, and CUC members) are a plant-specific super family and different members have a variety of important functions. Most NAC proteins contain a highly conserved N-terminal DNA-binding domain, and a variable C-terminal domain^20^. The tomato genome contains 101 NAC TFs^21^. In addition to the famous *NOR*^6,22^, two other NAC family genes *SlNAC1* and *SlNAC4* have been shown to be involved in the regulation of tomato fruit ripening^23–25^. In *SlNAC1*-overexpressing fruits, ethylene production was decreased, fruit softened earlier and remained yellow or orange when fully ripe^23^. In *SlNAC1*-RNAi transgenic lines, although ethylene production was delayed, it eventually reached a higher level than that in WT^24^. Reduced *SlNAC4* expression by RNAi delayed fruit ripening by 2–3 days^25^. Despite the large number of NAC genes in plants, there is no information about the possible role of other NAC family members associated with fruit ripening, and the precise target genes of NOR, SlNAC1, and SlNAC4 are still unclear.

In this report, we used TRV-mediated VIGS to screen NAC TFs candidates and found that silencing *NOR-like1* (Solyc07g063420), markedly suppressed tomato fruit ripening. We also obtained two stable knock-out mutants of *nor-like1* by CRISPR/Cas9 and they showed a similar phenotype to the VIGS*-NOR-like1* silenced fruit. Further study showed that NOR-like1 directly binds to the promoters of several genes involved in tomato ripening processes, including ethylene biosynthesis, color change, and cell wall metabolism, and positively regulates their expression. Our data define important NOR-like1 targets and establish the role of NOR-like1 as a new positive regulator of tomato fruit ripening.

## Results

### Virus-induced gene silencing of *NOR-like1* delayed tomato fruit ripening

**Fig. 1:**
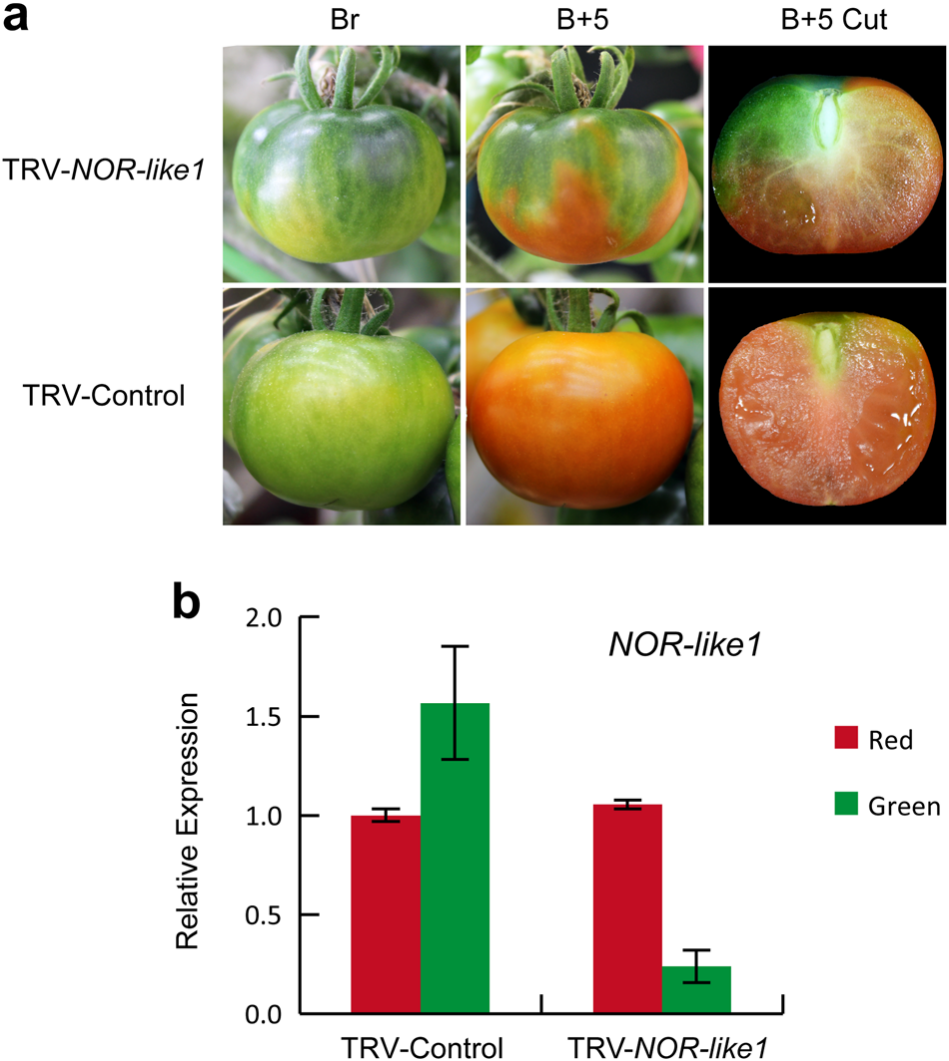
Virus-induced gene silencing of *NOR-like1* delays tomato fruit ripening. **a** Virus-induced gene silencing assay in wild-type (WT) tomato fruits (*Ailsa Craig*, AC) shows the effect of *NOR-like1* on fruit ripening. Br, breaker; B + 5, 5 days after breaker. **b** qRT-PCR analysis of *NOR-like1* transcripts in TRV-*NOR-like1* and TRV-control tomato fruits at B + 5 stage. *Actin* was used as the internal control. Values are means ± SD of three independent replicates

In a systematic study of NAC TF genes, potentially related to tomato fruit ripening, we obtained a striking inhibition of ripening in VIGS fruits infected with a TRV-*NOR-like1* (Solyc07g063420) construct. Fruit showed mottled green and orange areas, separated by a distinct border, which contrasted with the uniform orange phenotype observed in the control fruit at B + 5 (5 days after color break) (Fig. 1a), suggesting that *NOR-like1* may be involved in regulating tomato fruit ripening. Quantitative reverse transcription polymerase chain reaction (qRT-PCR) result showed that the *NOR-like1* transcripts in the green sections of TRV-*NOR-like1*-infected fruit were significantly lower than in the orange sections and the green and orange stage of the control fruits infected by TRV alone (Fig. 1b), confirming that *NOR-like1* gene silencing was associated with the uneven color phenotype.

### NOR-like1 is located in the nucleus and highly expressed in tomato fruit

Phylogenetic analysis of NOR-like1 and other NAC proteins in *Arabidopsis* and tomato showed that NOR-like1 and NOR (sometimes referred to NAC-NOR) are closely related (Supplemental Figure S1). The *NOR-like1* gene is 2811 bp long, comprises three exons and two introns, encodes a protein of approximately 37 KD (329 amino acid), with a conserved NAC domain at the N-terminus and an un-conserved transcriptional activation domain at the C-terminus (Supplemental Figure S2), all typical features of the NAC TF family.

**Fig. 2:**
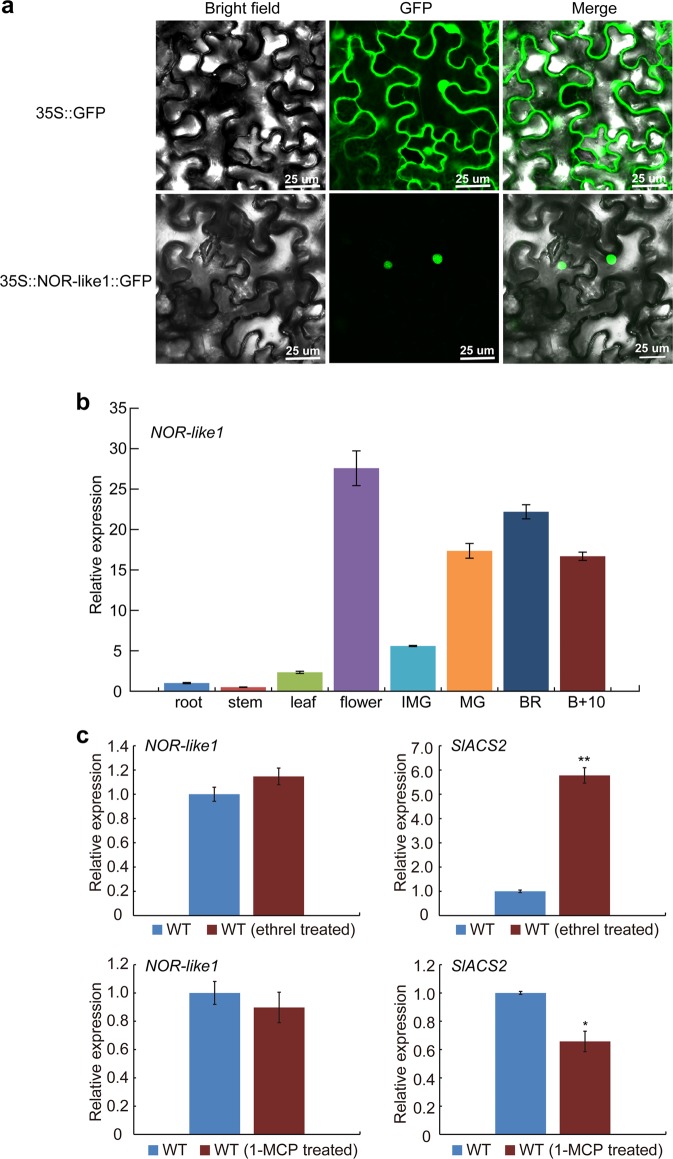
Subcellular localization of NOR-like1 in nuclei, gene expression pattern of *NOR-like1* and the expression of *NOR-like1* in WT fruit after treatment with ethrel and 1-MCP. **a** Subcellular localization of NOR-like1 in nuclei. Tobacco leaves were used for subcellular localization. Green fluorescence images were taken in a dark field, while the outline of the cell was photographed in a bright field. 35S:NOR-like1:GFP represents NOR-like1 and GFP fusion protein. 35S:GFP represents the control. Bars = 25 μm. **b** qRT-PCR analyses of *NOR-like1* in different tomato organs and fruit ripening stages. IMG, immature green. *Actin* was used as the internal control. Bars represent ± SD of three independent replicates. **c** Expression of *NOR-like1* in WT fruit after treated with ethrel and 1-MCP. *Actin* was used as the internal control. *SlACS2* was detected as the positive control. Error bars indicate ± SD of three biological replicates. Asterisks indicate significant differences determined by Student’s *t*-test (**p < 0.01, *p < 0.05)

Subcellular localization of NOR-like1 protein clearly indicated that the NOR-like1:GFP fusion protein was exclusively localized in the nucleus (Fig. 2a), consistent with a putative role in transcriptional regulation.

During tomato plant growth and development, *NOR-like1* transcripts were present in much lower amounts in vegetative organs such as roots, stems, and leaves, but were highly expressed in reproductive organs such as flowers and fruits. During fruit ripening, the expression of *NOR-like1* increased rapidly from the mature green (MG) stage and reached the highest expression level at the breaker stage (Fig. 2b). But the expression of *NOR-like1* was not affected by exogenous ethylene or the ethylene perception inhibitor 1-Methylcyclopropene (1-MCP) (Fig. 2c). The expression pattern and insensitivity to ethylene suggested that *NOR-like1* may have an active role in fruit ripening initiation or development.

### *NOR-like1* CRISPR/Cas9-edited tomato fruits fail to produce NOR-like1 protein and ripening is significantly inhibited

**Fig. 3:**
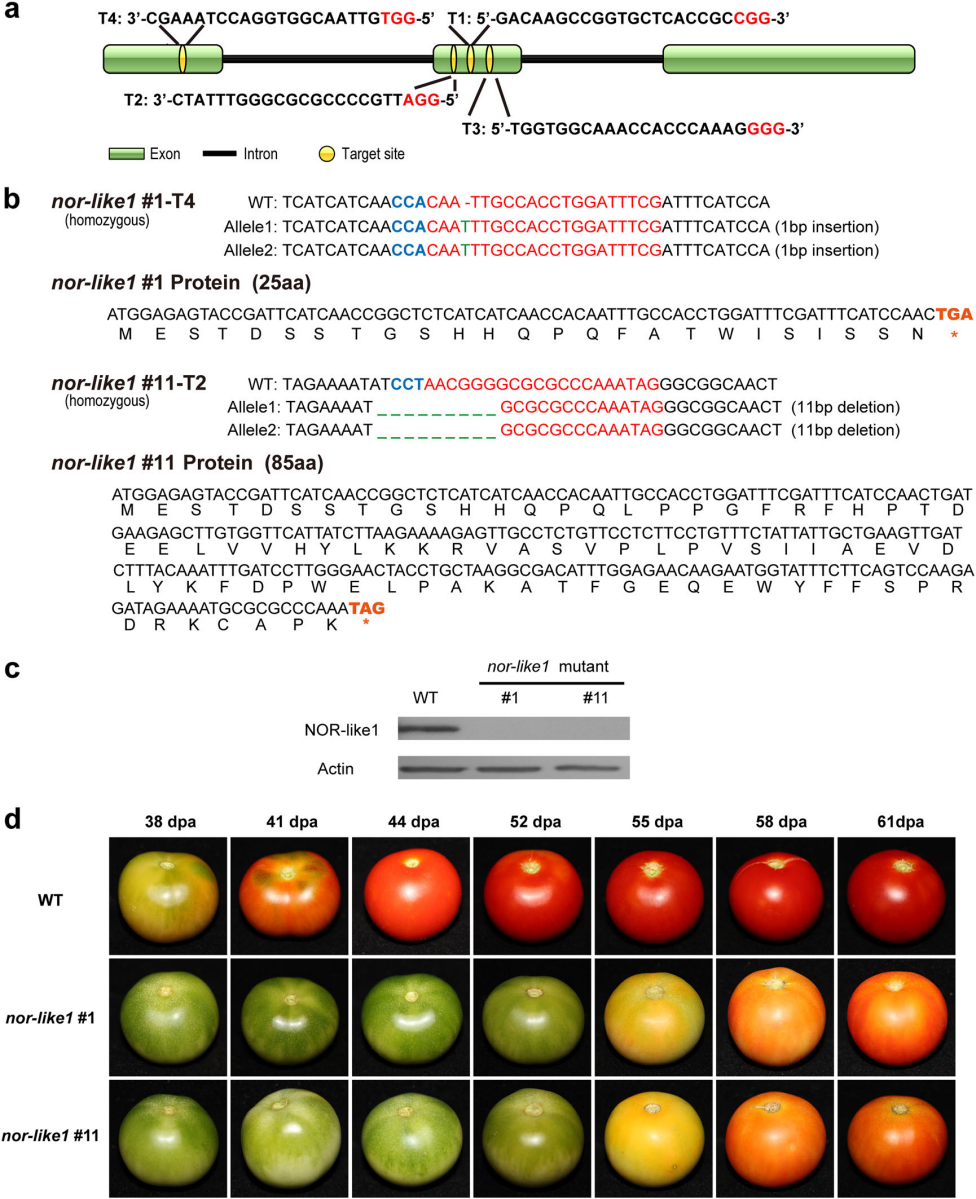
Tomato fruit ripening was greatly delayed in *nor-like1* mutants. **a** Schematic illustration of four target sites in *NOR-like1* genomic sequence. **b** Gene editing analysis of *nor-like1*#1 and *nor-like1*#11 mutants. Red letters indicate the target sites, green letters represent edited site and editing type, and blue letters represent the protospacer adjacent motif (PAM). **c** The absence of antibody-detectable NOR-like1 protein in *nor-like1*#1 and *nor-like1*#11 mutants. Actin was used as the internal control. **d** Tomato fruit ripening was greatly delayed in *nor-like1#1* and *nor-like1#11* mutants compared with that in WT. Dpa, days post anthesis

To gain insight into the function of *NOR-like1* in tomato fruit, we generated *NOR-like1* knock-out mutants in tomato cultivar *Ailsa Craig* (AC) using CRISPR/Cas9 gene editing technology. In total, sixteen independent T_0_ transgenic lines were produced (Supplemental Figure S3a). Of these lines, six were genome edited and the editing rates of each target sites are shown in Supplemental Figure S3b. We selected two representative T_0_ transgenic lines (*CR-NOR-like1#1* and *CR-NOR-like1#11*) for further analysis (the editing details of the two lines are shown in Supplemental Figure S3c). In the two lines, the average number of days from anthesis to the breaker stage of ripening was increased and the rate of ripening after the breaker stage was inhibited (Supplemental Figure S4). Then we obtained two homozygous lines from *CR-NOR-like1#1* and *CR-NOR-like1#11*, named *nor-like1*#1 and *nor-like1*#11, respectively (details shown in Fig. 3b). To confirm the premature termination of NOR-like1 protein translation, the expression levels of NOR-like1 protein in *nor-like1#1*, *nor-like1#11*, and WT tomato fruit at the breaker stage were examined by western blot using NOR-like1-specific antibody produced from the C-terminus of NOR-like1 protein. The results showed that the mature NOR-like1 protein was absent from *nor-like1#1* and *nor-like1*#*11* mutants compared with that in WT fruit (Fig. 3c), suggesting that these two *nor-like1* mutant lines were prematurely terminated at the N-terminus of the NOR-like1 protein.

To test for off-target gene editing in *nor-like1#1* and *nor-like1#11* mutants, two of the most likely off-target sites were tested (Supplemental Table S7) and the results showed that no off-target activity occurred in either of the two lines. Furthermore, we examined whether there was editing in the genomic DNA of *NOR* (Solyc10g006880), *SlNAC4* (Solyc11g017470), and *SlNAC1* (Solyc04g009440), which have been reported to have an important role in tomato fruit ripening^6,22–25^. The results indicated that the genomic DNA sequences of the three related genes were all unedited (Supplemental Figure S5, S6, and S7), confirming that *nor-like1#1* and *nor-like1#11* mutants only had the *NOR-like1* gene editing.

In order to analyze the ripening phenotype of *nor-like1* mutants, the fruit ripening process in mutants and WT were studied in detail. The results showed that ripening in both *nor-like1#1* and *nor-like1#11* lines was markedly inhibited and fruit reached the breaker stage 14 days or more later than WT. In addition, fruits failed to turn fully red and remained an orange-red color at the final ripe stage (Fig. 3d). The results verified the fruit phenotypes generated from the *NOR-like1*-VIGS silenced tomato plants (Fig. 1a), suggesting that NOR-like1 regulates tomato fruit ripening. When we tried to harvest seeds for reproduction, however, we were surprised to find that the *NOR-like1* mutation seriously affected seed development (Supplemental Figure S8a), reducing the number (by 78% and 74.8%, respectively) and weight (by 54.9% and 61.1%, respectively) of seeds (Supplemental Figure S8b) and the remaining seeds showed poor germination.

### NOR-like1 alters expression of key genes involved in tomato fruit ripening

**Fig. 4:**
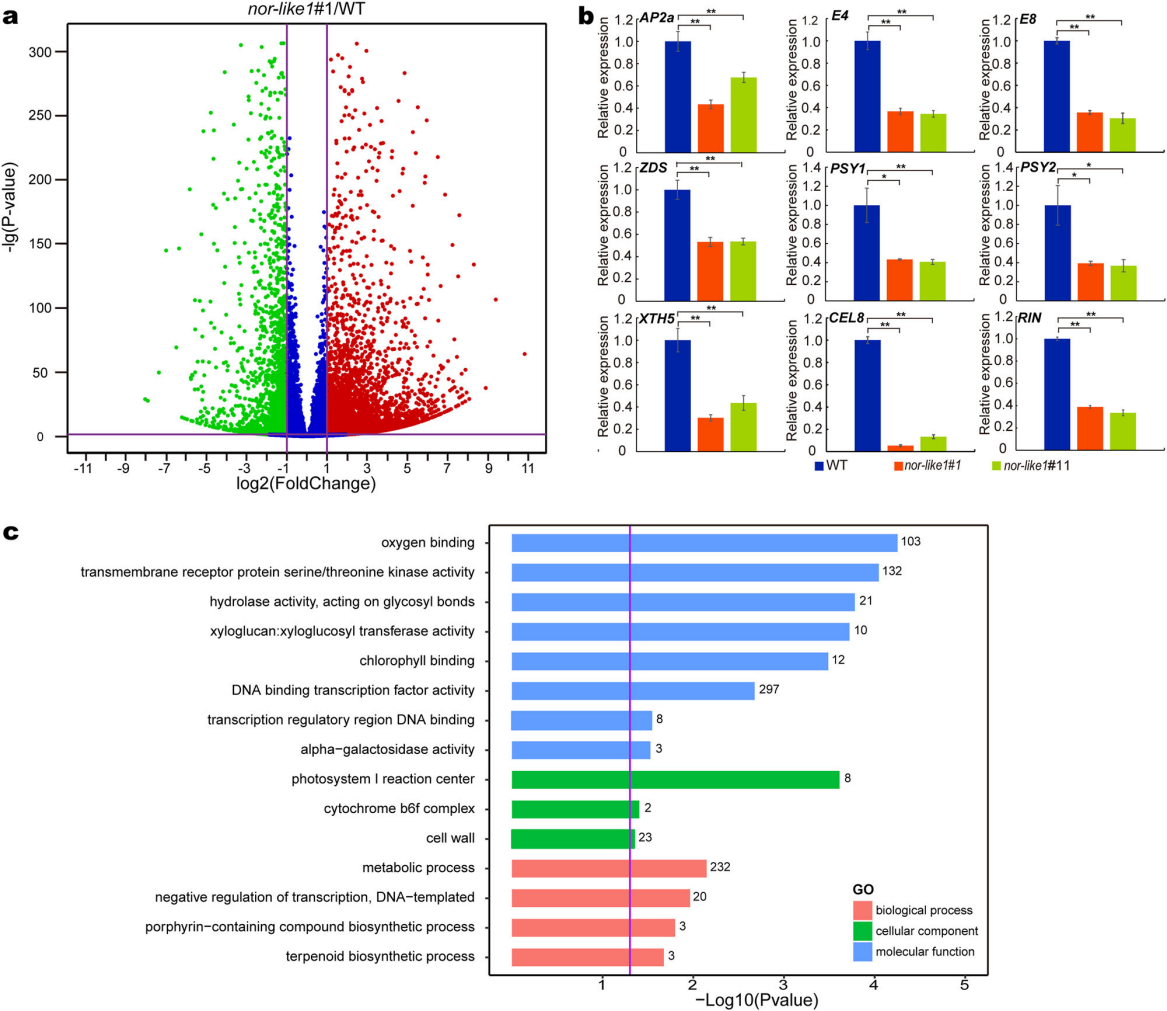
*NOR-like1* affects the expression of genes related to tomato fruit ripening. **a** RNA-seq data visualized by volcano plots. Each point represents a DEG. Red points represent upregulated genes, and green points represent downregulated genes (*nor-like1*#1/WT). | Log2 (fold change) | = 1 and *p*-value = 0.05 are marked with purple lines. **b** Validation of RNA-seq results by qRT-PCR. Nine genes of different expression levels were selected to be tested by qRT-PCR in *nor-like1#1*, *nor-like1#11* and WT fruits at the B + 3 stage. *Actin* gene was used as the internal control. Bars represent ± SD of three independent replicates. **c** GO functional enrichment analysis of genes different in abundance between *nor-like1#1* and WT fruits at B + 3 stage. *p*-value = 0.05 was marked with a purple line

To understand how *NOR-like1* mutation affects tomato ripening at the molecular level, RNA-sequencing (RNA-Seq) profiling was performed to evaluate the effect of loss function of NOR-like1 on the entire transcriptome. The reads per kilobase per million mapped reads (RPKM) values of three biological replicates for each sample were highly correlated, indicating that the RNA-seq data were reliable (Supplemental Figure S9a). On the basis of a cutoff threshold of | Log2 (fold change) | > 1 and *p*-value < 0.05, analysis of differentially expressed genes (DEGs) revealed that 4453 genes were upregulated while 2254 genes were downregulated in the *nor-like1#1* mutant compared with that in WT (Fig. 4a; Supplemental Data Set S1; Supplemental Figure S9b). The reliability of the RNA-Seq data was tested by examining the transcript level of 9 genes (*SlAP2a; SlE4; SlE8; SlZDS; SlPSY1; SlPSY2; SlXTH5; SlCEL8*, and *SlRIN*) in *nor-like1#1*, *nor-like1#11* and WT by qRT-PCR, the results were highly correlated with the RNA-seq data (*R*^2^ = 0.91) (Fig. 4b; Supplemental Figure S10). Gene ontology (GO) analysis indicated that loss of NOR-like1 function affected multiple metabolic pathways, including the cell wall, xyloglucosyl transferase activity, chlorophyll binding, cytochrome b6f complex, DNA-binding transcription factor activity, porphyrin-containing compound biosynthetic process, and terpenoid biosynthetic process (Fig. 4c). Further analysis of DEGs showed that a series of key genes involved in the ethylene biosynthesis and signal transduction pathway, carotenoid biosynthesis pathway, softening pathway, and some crucial TFs were also affected by *NOR-like1* gene editing (Table 1). The data suggest that NOR-like1 regulates tomato fruit ripening by regulating the transcription of ripening-associated genes.

**Table 1:** Gene expression changes involved in tomato fruit ripening between *nor-like1* mutant and WT fruits at the B + 3 stage

Gene names	Gene ID	Log2 (fold change)	Annotation
*ACS2*	Solyc01g095080	−3.65	1-aminocyclopropane-1-carboxylate synthase
*ACS4*	Solyc05g050010	−2.73	1-aminocyclopropane-1-carboxylate synthase
*AP2a*	Solyc03g044300	−1.88	AP2-like ethylene-responsive transcription factor
*E4*	Solyc03g111720	−1.19	Peptide methionine sulfoxide reductase msrA
*E8*	Solyc09g089580	−1.96	1-aminocyclopropane-1-carboxylate oxidase-like protein
*DXS*	Solyc01g067890	−1.21	1-deoxy-D-xylulose 5-phosphate synthase 1
*Ggpps2*	Solyc04g079960	−3.03	Geranylgeranyl pyrophosphate synthase 2
*PSY1*	Solyc03g031860	−1.13	Phytoene synthase 1
*PSY2*	Solyc02g081330	−1.53	Phytoene synthase 2
*PDS*	Solyc03g123760	−1.06	Phytoene_desaturase
*ZDS*	Solyc01g097810	−1.17	Zeta-carotene desaturase
*SGR1*	Solyc08g080090	−4.20	Senescence-inducible chloroplast stay-green protein 2
*PL*	Solyc03g111690	−3.23	Pectate lyase
*EXP1*	Solyc06g051800	−5.31	Expansin
*CEL2*	Solyc09g010210	−10.13	Endo-glucanase
*PG2a*	Solyc10g080210	−4.17	Polygalacturonase A
*CEL8*	Solyc08g082250	−4.61	Endo-glucanase
*XTH5*	Solyc01g081060	−2.00	Xyloglucan endotransglucosylase/hydrolase 14
*RIN*	Solyc05g012020	−2.10	MADS-box transcription factor
*TDR4/FUL1*	Solyc06g069430	−1.13	MADS-box transcription factor
*TAGL1*	Solyc03g123760	−1.06	Agamous MADS-box transcription facto
*ZFP2*	Solyc07g055920	−1.36	Zinc-finger protein 1

### NOR-like1 binds to the promoters of *SlACS2* and *SlACS4* to regulate ethylene production

**Fig. 5:**
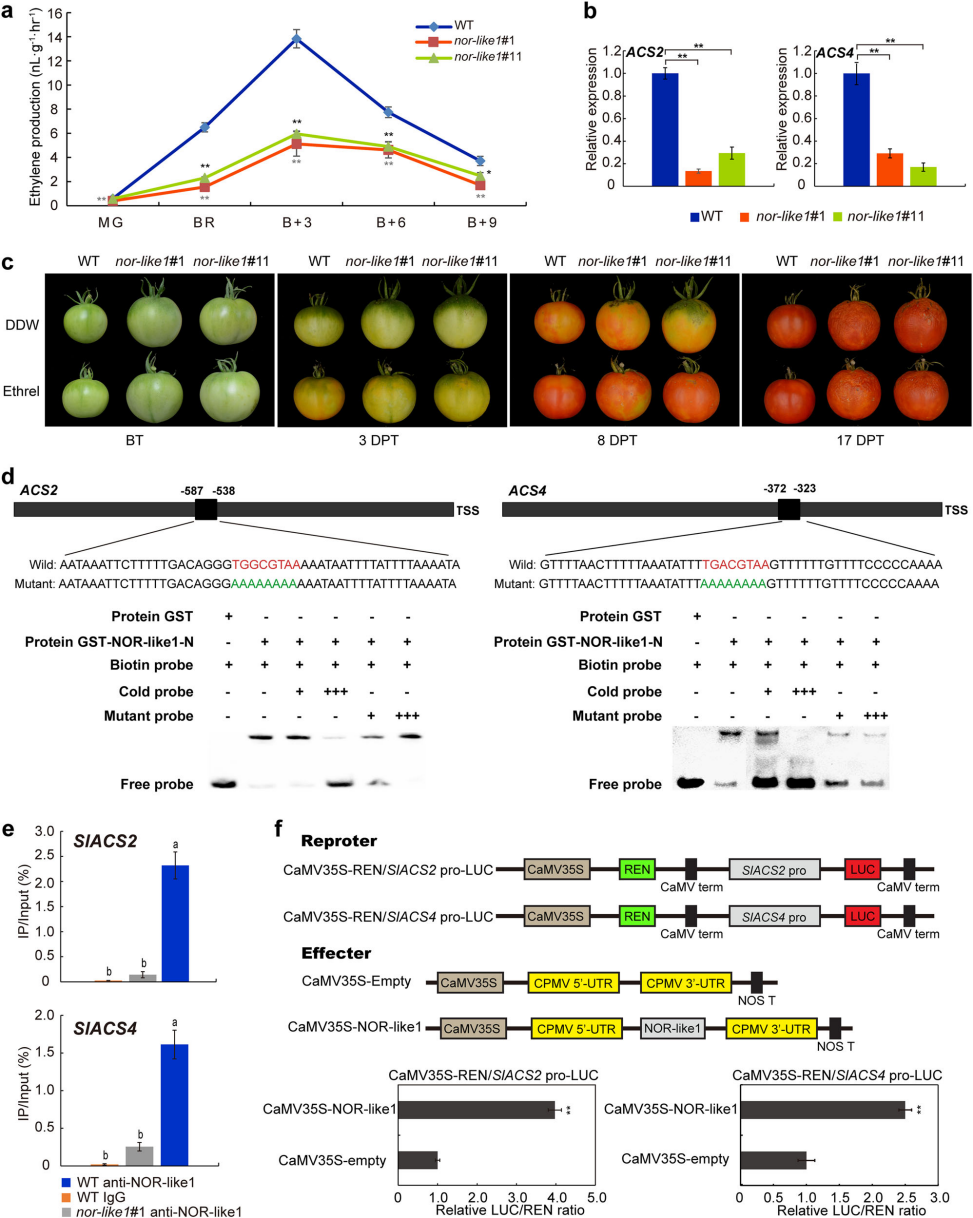
*SlACS2* and *SlACS4* are directly regulated by NOR-like1 resulting in the reduction of ethylene production in CRISPR/Cas9 *nor-like1* mutant fruits. **a** Ethylene production was reduced in *nor-like1*#1 and *nor-like1*#11 mutants compared with that in WT. Asterisks indicate significant difference determined by Student’s *t*-test (*p < 0.05; **p < 0.01), asterisks marked in dark gray indicated significant difference of *nor-like1*#1 and WT, the significant difference of *nor-like1*#11 and WT were marked in black. **b** Expression of *SlACS2* and *SlACS4* was significantly downregulated both in *nor-like1*#1 and *nor-like1*#11 mutants compared with that in WT. Asterisks indicate *p* < 0.01 (Student’s *t*-test). **c** The phenotype of WT and *nor-like1*#1 and *nor-like1*#11 mutant fruits after treatment with ethrel. DDW, double distilled water, BT before treatment, DPT, days post treatment. **d** Binding of NOR-like1 to the promoters of downstream target genes *SlACS2* and *SlACS4*. The sequences of the wild-type probes containing the NACRS were biotin-labeled. Competition for NOR-like1 binding was performed with 50× and 500 ×  cold probes containing the wild-type NACRS (indicated with red letters) or mutated NACRS (indicated with green letters). The symbols + or – represent presence or absence, respectively, +++ indicates increasing amounts. **e** ChIP-qPCR assay showing the direct binding of NOR-like1 to the promoters of *SlACS2* and *SlACS4*. Values represent the percentage of DNA fragments in WT fruits that co-immunoprecipitated with anti-NOR-like1 or IgG, or DNA fragments in *nor-like1*#1 fruit that co-immunoprecipitated with anti-NOR-like1 relative to the input DNA. Bars represent ± SD of three independent replicates. The lowercase indicates significant difference (Duncan’s multiple range test, *p* < 0.05). **f** Transient expression assay for NOR-like1 activation of the *SlACS2* and *SlACS4* promoters. Each value represents the means of three biological replicates. Asterisks indicate *p* < 0.01 (Student’s *t*-test)

Ethylene has a key role in the regulation of climacteric fruits ripening process^22^. To test whether NOR-like1 regulates ethylene biosynthesis in tomato fruit, gas chromatography (GC) analysis of ethylene evolution from WT and *nor-like1* mutant fruits was performed. We found that ethylene production by the fruit of both *nor-like1#1* and *nor-like1#11* lines was approximately halved compared with that in WT during fruit ripening (Fig. 5a). Furthermore, qRT-PCR analysis showed that the expression of *SlACS2* and *SlACS4* genes was markedly reduced by between 70-80% both in *nor-like1#1* and *nor-like1#11* lines compared to WT (Fig. 5b). To find out whether the inhibited ripening phenotype of *nor-like1* mutants could be restored by supplying exogenous ethylene, we treated the mature green *nor-like1* mutant and WT fruits with the ethylene-generating compound ethrel (0.4%). Although the maturation process was promoted, the fruit of *nor-like1#1* and *nor-like1#11* mutants still could not become fully red like WT (Fig. 5c). To understand whether NOR-like1 directly regulates transcription of *SlACS2* and *SlACS4* during tomato fruit ripening, we performed promoter analysis of *SlACS2* and *SlACS4* genes and found that there was a NAC recognition sequence (NACRS, [TA][TG][AGC]CGT[GA][TA]) containing the core CGT[GA] motif^26^ in the 2 -kb upstream regions of the promoters of both genes. To test whether NOR-like1 protein directly binds the promoter regions of *SlACS2* and *SlACS4* genes, EMSA was performed. The results showed that NOR-like1 could specifically bind to the NACRS motif in the *SlACS2* and *SlACS4* promoters (Fig. 5d). ChIP-qPCR results showed that NOR-like1 could directly bind to the *SlACS2*/*SlACS4* promoters in tomato fruit (Fig. 5e). In *nor-like1* mutants, the expression of *SlACS2* and *SlACS4* was significantly inhibited, suggesting that NOR-like1 protein positively regulated the transcription of both genes. To test this assumption, DLR assay was performed. The relative LUC/REN ratio in tobacco leaves co-transformed with CaMV35S-NOR-like1 and CaMV35S-REN/p*SlACS2-*LUC or CaMV35S-REN/p*SlACS4*-LUC was significantly higher than in leaves co-transformed with CaMV35S-Empty and CaMV35S-REN/p*SlACS2*-LUC or CaMV35S-REN/p*SlACS4*-LUC (Fig. 5f), indicating that NOR-like1 could activate the promoter activity of *SlACS2* and *SlACS4* in tobacco. Taken together, the results demonstrated that NOR-like1 is a transcriptional activator that positively regulates ethylene biosynthesis in tomato fruit by directly targeting the promoter of *SlACS2* and *SlACS4*.

### NOR-like1 positively regulates the expression of *SlGgpps2* and *SlSGR1* involved in color change in ripening tomato fruits

**Fig. 6:**
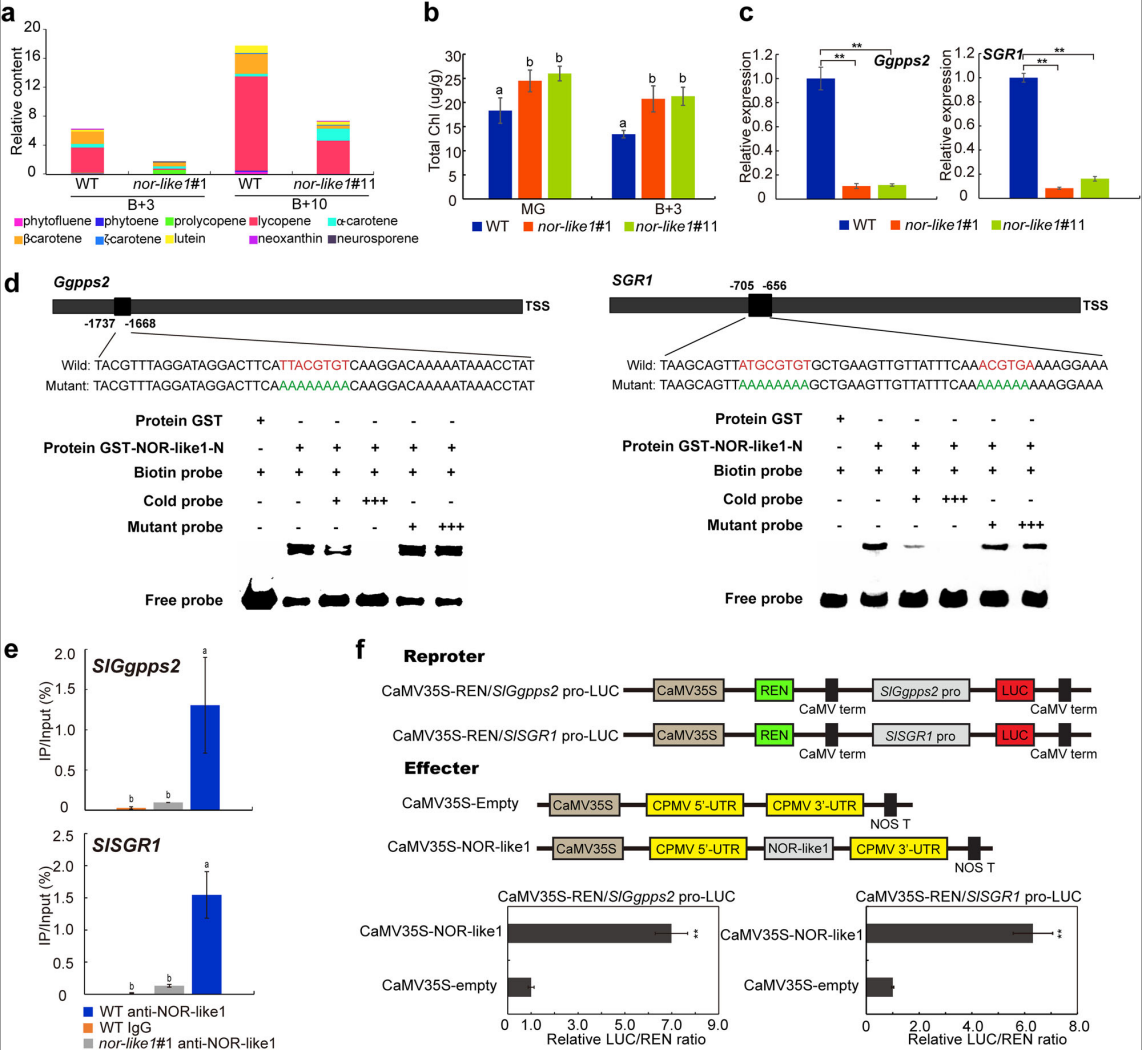
*SlSGR1* and *SlGgpps2* are directly regulated by NOR-like1, carotenoid accumulation is reduced, and chlorophyll degradation is inhibited in CRISPR/Cas9 *nor-like1* mutants. **a** Carotenoid accumulation was reduced in tomato fruits of *nor-like1#1* mutant at B + 3 and B + 10 ripening stages compared with that in WT. **b** Total chlorophyll content in *nor-like1#1* and *nor-like1#11* mutants was greater than that of in WT at MG and B + 3 stages. **c** Expression of *SlGgpps2* and *SlSGR1* were significantly downregulated both in *nor-like1#1* and *nor-like1#11* mutants compared with that in WT. Asterisks indicate *p* < 0.01 (Student’s *t*-test). **d** Binding of NOR-like1 to the promoters of downstream target genes *SlGgpps2* and *SlSGR1*. The sequences of the wild-type probes containing the NACRS were biotin-labeled. Competition for NOR-like1 binding was performed with 50× and 500 ×  cold probes containing the wild-type NACRS (indicated with red letters) or mutated NACRS (indicated with green letters). The symbols + or – represent presence or absence respectively. +++ indicates increasing amounts. **e** ChIP-qPCR assay showing the direct binding of NOR-like1 to the promoter of *SlGgpps2* and *SlSGR1*. Values represent the percentage of DNA fragments in WT fruits that co-immunoprecipitated with anti-NOR-like1 or IgG, or DNA fragments in *nor-like1#1* fruit that co-immunoprecipitated with anti-NOR-like1 relative to the input DNA. Bars represent ± SD of three independent replicates. Significant differences (Duncan’s multiple range test, *p* < 0.05) are indicated in lowercase. **f** Transient expression assay of *NOR-like1* strongly activated the *SlGgpps2* and *SlSGR1* promoters. Each value is the mean of three biological replicates. Asterisks indicate *p* < 0.01 (Student’s *t*-test)

The process of tomato fruit ripening is accompanied by chlorophyll degradation and lycopene accumulation, which is a transcriptionally coordinated regulatory process^27^. To evaluate the characteristics of tomato fruit color change, carotenoids were measured at the B + 3 and B + 10 stages of *nor-like1#1* mutant and WT fruits. The results showed that the level of *β*-carotene and lycopene decreased significantly in *nor-like1* mutant fruit compared with that in WT fruit (Fig. 6a). In addition, we also found that the degradation rate of chlorophyll in the mutants was significantly lower than in WT. Measurement of the total chlorophyll content of *nor-like1* mutants and WT at MG and B + 3 stages confirmed that the total chlorophyll content of *nor-like1* mutants was higher than that of the control fruit (Fig. 6b). RNA-seq data indicated that *SlGgpps2* (encoding geranylgeranyl pyrophosphate synthase 2) and *SlSGR1* (encoding senescence-inducible chloroplast stay-green protein 2), which are involved in carotenoid accumulation and chlorophyll degradation, respectively, were markedly downregulated, and promoter analysis suggested that both genes contain a NACRS motif in the promoter region, so we speculated that these two genes could be NOR-like1 targets. Analysis of qRT-PCR confirmed that *SlGgpps2* and *SlSGR1* expression was markedly inhibited in *nor-like1* mutant fruit compared with that in WT at B + 3 stage (Fig. 6c) and results of EMSA (Fig. 6d) and ChIP-qPCR (Fig. 6e) confirmed that NOR-like1 was able to bind to the *SlGgpps2* and *SlSGR1* promoters both in vitro and in vivo. Furthermore, transient expression analysis demonstrated that NOR-like1 could activate the promoter of *SlGgpps2 and SlSGR1* in tobacco leaves (Fig. 6f). Taken together, our data suggest that NOR-like1 regulates the color change of tomato fruit by directly upregulating the transcription of *SlGgpps2* and *SlSGR1*.

### NOR-like1 directly regulates expression of *SlPG2a*, *SlPL*, *SlCEL2* and *SlEXP1* and affects the firmness of tomato fruits

**Fig. 7:**
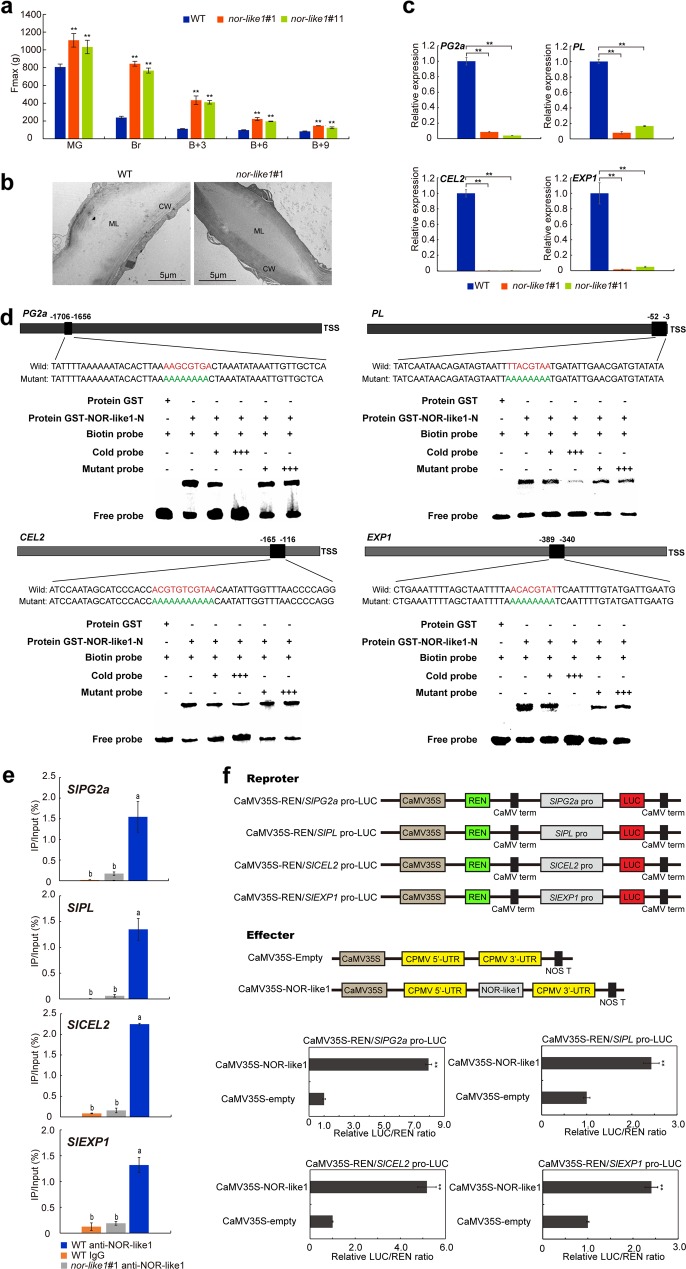
*SlPG2a*, *SlPL*, *SlCEL2*, and *SlEXP1* are directly regulated by NOR-like1 resulting in increased tomato fruit firmness in *nor-like1* mutants. **a** The fruit softening process was delayed and softening degree was reduced in *nor-like1* mutant tomato fruits compared with that in WT fruits. **b** TEM indicating that the *nor-like1* mutant fruit cell walls were more deeply stained and had more closely arranged microfilaments and dark middle lamella compared with that in WT fruits. CW, cell wall, ML, middle layer. Bars = 5 μm. **c** Expression of *SlPG2a, SlPL, SlCEL2,* and *SlEXP1* was significantly downregulated both in *nor-like1*#1 and *nor-like1*#11 mutants compared with that in WT. Asterisks indicate *p* < 0.01 (Student’s *t*-test). **d** Binding of NOR-like1 to the promoters of downstream target genes *SlPG2a, SlPL, SlCEL2*, and *SlEXP1*. The sequences of the wild-type probes containing the NACRS were biotin-labeled. Competition for NOR-like1 binding was performed with 50× and 500 × cold probes containing the wild-type NACRS (indicated with red letters) or mutated NACRS (indicated with green letters). The symbols + or − represent presence or absence, respectively, +++ indicates increasing amounts. **e** ChIP-qPCR assay showing the direct binding of NOR-like1 to the promoter of *SlPG2a, SlPL, SlCEL2*, and *SlEXP1*. Values represent the percentage of DNA fragments in wild-type fruits that co-immunoprecipitated with anti-NOR-like1 or IgG, or DNA fragments in *nor-like1#1* fruit that co-immunoprecipitated with anti-NOR-like1 relative to the input DNA. Bars represent ± SD of three independent replicates. Significant differences (Duncan’s multiple range test, *p* < 0.05) are indicated in lowercase. **f** Transient expression assay showing NOR-like1 greatly activated the *SlPG2a, SlPL, SlCEL2,* and *SlEXP1* promoters. Each value represents the means of three biological replicates. Asterisks indicate *p* < 0.01 (Student’s *t*-test)

The degradation of the cell wall leads to fruit softening during ripening^28,29^. To evaluate the effect of the *NOR-like1* mutation on tomato fruit softening, the fruit firmness of WT and *nor-like1* mutant fruits at five ripening stages (MG, Br, B + 3, B + 6, B + 9) were measured by texture analyzer. The fruit firmness of *nor-like1#1* and *nor-like1#11* mutant fruits was significantly higher than that in WT fruit at all five ripening stages examined (Fig. 7a). Furthermore, transmission electron microscopy (TEM) indicated that the cell walls of the *nor-like1* mutant were dark-colored with a dark middle lamella and microfilaments more closely arranged compared with that in the WT cell walls (Fig. 7b), suggesting that cell wall degradation was reduced in the fruit of *nor-like1* mutant. To search for cell wall associated genes directly regulated by NOR-like1, we screened the RNA-seq DEGs to obtain the genes related to cell wall metabolism, and then analyzed their 2-kb promoter regions to detect NACRS motif. The fact that the expression levels of 4 genes related to cell wall metabolism, polygalacturonase 2a (*SlPG2a*), pectate lyase (*SlPL*), Endo-glucanase 2 (*SlCEL2*), and expansin1 (*SlEXP1*) were downregulated by at least 89% (Fig. 7c) and the promoter regions of these four genes all contain the NACRS motif suggested they could be NOR-like1 targets. The results from EMSA analysis (Fig. 7d), ChIP-qPCR (Fig. 7e) and DLR (Fig. 7f) experiments all demonstrated that NOR-like1 could bind to *SlPG2a*, *SlPL*, *SlCEL2* and *SlEXP1* promoters in vitro and in vivo and activate their expression, suggesting that NOR-like1 positively regulates cell wall metabolism by targeting *SlPG2a*, *SlPL*, *SlCEL2*, and *SlEXP1*.

## Discussion

### NOR-like1 and NOR proteins have homology but low functional redundancy

NOR-like1 has 62.84% amino acid homology to NOR, which is a global ripening regulator^22^, and they belong to a small branch of the NAC phylogenetic tree (supplemental Figure S1). Despite this, the two mutant phenotypes are different; *nor* mutant fruits produce no ethylene and remain green^6^, whereas mutant fruit of *nor-like1*, although showing an obvious delay in ripening (Fig. 3d), could produce some ethylene and carotenoids but the ripe fruits remained orange-red. In addition, we found that seed development in *nor-like1* mutant fruit was defective (supplemental Figure S8), whereas the *nor* mutant is known to produce normal seeds. Previous research also showed that *NOR-like1*-RNAi transgenic lines had reduced seed size^30^. In *Arabidopsis*, seed development is controlled by two homologous genes *NARS1* (ANAC056, AT3G15510) and *NARS2* (ANAC018, AT1G52880)^31^, and their proteins have the closest evolutionary relationship with NOR-like1 and NOR (Supplemental Figure S1). In tomato, however, it seems that only *NOR-like1* but not *NOR* controls seed development. These results indicate that *NOR-like1* and *NOR* may have some overlapping but also different functions in the regulation of tomato fruit ripening and seed development.

### NOR-like1 regulates ethylene biosynthesis but is not involved in ethylene feedback regulation

**Fig. 8:**
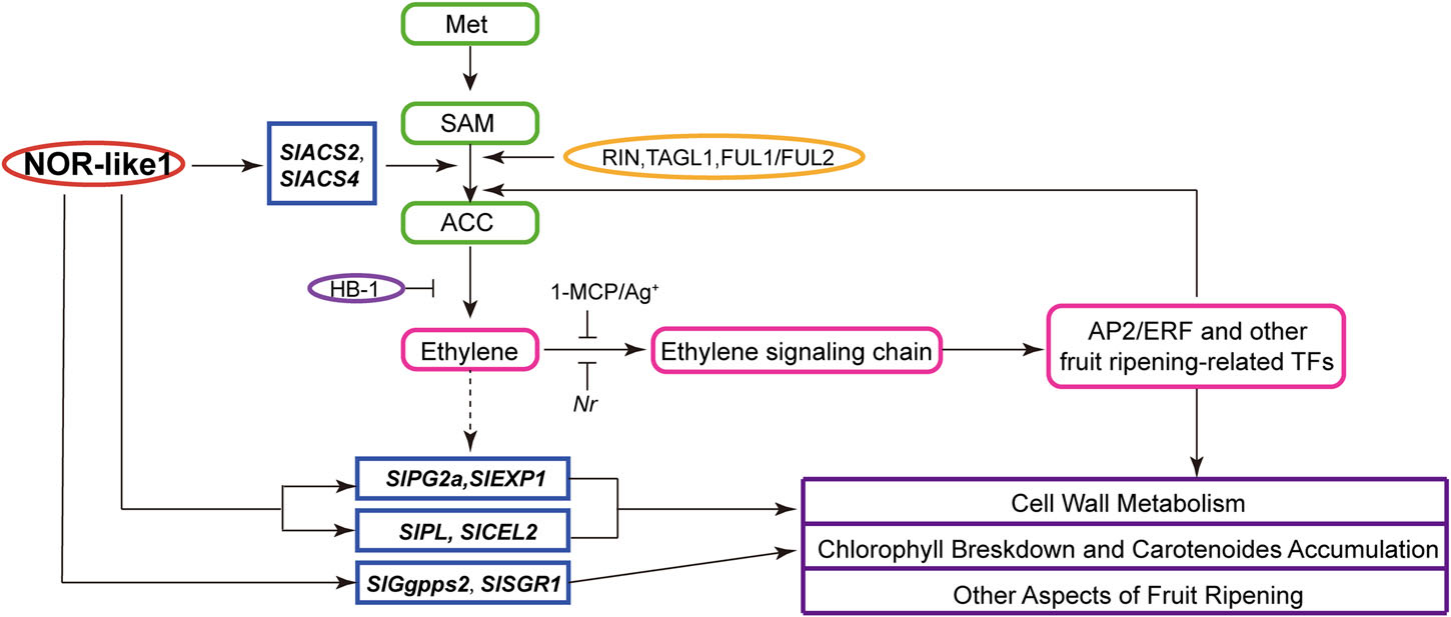
Working model of the role of NOR-like1 in the tomato fruit ripening control network and expression of ripening-associated genes

Tomato is classified physiologically as a climacteric fruit, based on the marked induction of respiration and ethylene production at the onset of ripening. Ethylene synthesis is tightly controlled during the plant life cycle and two modes (System-1 and System-2) of ethylene regulation have been proposed^32–34^. Transition from System-1 to System-2 ethylene during the onset of ripening is correlated with increased ACC synthase (*SlACS1A*, *SlACS2*, and *SlACS4*) expression^33,35,36^. *SlACS2* is largely ethylene-inducible in mature fruit while both *SlACS2* and *SlACS4* are under additional developmental control^33^. ACC Oxidase (*SlACO1*, *SlACO3*), *SlACS2* and *SlACS4* are responsible for the production of System-2 ethylene during tomato fruit ripening^37^ and are regulated by ripening-associated TFs^38^. It has been shown previously that RIN forms complexes with TAGL1 and FUL1/FUL2 and regulates expression of ripening-related genes including *SlACS2* and *SlACS4*^15,38^. Here, we identified a new NAC TF, NOR-like1, that also positively regulates the expression of *SlACS2* and *SlACS4* (Figs. 5, 8) during tomato fruit ripening. This suggests that both RIN and NOR-like1 control ACC synthase genes *SlACS2* and *SlACS4*, but does not rule out the possibility of other interactions between RIN and NOR-like1.

Treating WT fruits with either exogenous ethylene or 1-MCP had no effect on the level of *NOR-like1* transcripts (Fig. 2c). This suggests that NOR-like1 acts on ethylene biosynthesis genes, but is not involved in the ethylene signal transduction pathway (Fig. 8). The *nor-like1* mutants could produce about 40% ethylene of the WT fruits (Fig. 5a). This is consistent with other TFs, such as RIN, also regulating expression of ethylene biosynthesis genes, and this may explain why the fruit ripening process in the mutants is only partially inhibited (Fig. 3d). The ripening deficiency of *nor-like1* mutants is not due simply to lack of ethylene since supplying ethylene externally did not restore full ripening. Although ethylene promoted ripening of *nor-like1* mutants to some extent, the ripe phenotype was not fully recovered, and the mutant fruit failed to turn completely red (Fig. 5c), suggesting that NOR-like1 protein has an important role in color development. These data suggest that expression of *NOR-like1* occurs at least partly independently of ethylene and that NOR-like1 shares the control of ethylene production with RIN, and perhaps other TFs (Fig. 8).

### NOR-like1 influences color change in ripening fruit via a direct effect on both chlorophyll degradation and carotenoid biosynthesis

Ripe tomato fruit color is determined by the accumulation of carotenoids, particularly lycopene, together with the concomitant degradation of chlorophylls, leading to marked color changes^39^, which are often used as an indicator of the extent of ripening and overall fruit quality. In higher plants, carotenoids are synthesized from IPP, which is converted to GGPP by IPP isomerase (IPI) and GGPP synthase (GGPPS)^40^, located upstream of the carotenoid synthesis pathway^39^. In *nor-like1* mutants, some genes (*DXS*, *Ggpps2*, *PSY1*, *PSY2*) involved in the carotenoid biosynthesis pathway were downregulated, the accumulation of carotenoids in mutant fruit was inhibited and the fruit finally exhibited an orange-red phenotype. Further analysis showed that NOR-like1 could bind to the promoter of *Ggpps2* and positively regulate its expression (Fig. 6d–f).

Fruit of *nor-like1* mutant remained green longer than WT, and it is particularly noteworthy that the process of fruit color transformation was slower after the breaker stage and degradation of chlorophyll was inhibited. The finding that *SlSGR1* is a direct target gene of NOR-like1 helps explain this phenotype. Previous discoveries have found that *SlSGR1* encodes a STAY-GREEN protein that has a critical role in the regulation of chlorophyll degradation in tomato leaves and fruits^41–43^. Silencing *SlSGR1* in tomato inhibits chlorophyll degradation and causes a stay-green phenotype^42^. *SlSGR1* can also regulate tomato lycopene accumulation through direct interaction with a key carotenoid synthetic enzyme *SlPSY1* and inhibit its activity^43^. This evidence and our results indicate that NOR-like1 controls color formation by directly regulating carotenoids (*SlGgpps2*) accumulation and chlorophyll metabolism (*SlSGR1*).

### NOR-like1 is a key regulator of tomato fruit softening

Fruit softening during ripening includes a series of modifications to the cell wall which consists of inextensible cellulose microfibrils held together by networks of hemicellulose and pectins, including attached structural glycoproteins and expansins^29,44^. Changes during ripening lead to pectin solubilization, cellulose and hemicellulose depolymerization, loosening of the xyloglucan-cellulose network and increased wall porosity caused by cell wall swelling, allowing greater access of degradative enzymes to their substrates^45^.

At least four enzymes have crucial roles in the process of pectin degradation during fruit ripening, polygalacturonase (PG), pectin methylesterase (PME), pectate lyase (PL), and β-galactosidase (TBG)^29^. PL breaks down cross-linked homogalacturonan polymers, particularly in tricellular junctions, PL and PG are involved in degradation of the pectic polysaccharides in the middle lamella^29,46^. Silencing PL was shown to increase fruit firmness without affecting other aspects of ripening, and the fruit shelf life was improved^46^. Previous studies also showed that tomato fruit firmness was increased by *TBG4*-inhibition^47^, but RNAi-mediated repression of either *PG2a* or *PME2* singly had little effect on the firmness of tomato fruit although it does affect pectin structure, and overexpression of *PG2a* in *rin* mutant could not restore fruit softening^48,49^. Our findings show that NOR-like1 is involved in the regulation of pectin dissolution during fruit softening by directly targeting *PL* and *PG2a*, leading to the inhibition of pectin solubilization in *nor-like1* mutant fruit.

During tomato fruit softening, the interaction between hemicellulose and cellulose becomes relaxed, which contributes to cell wall loosing. Xyloglucan endotransglycosylase (XTH) and endo-glucanase (CEL) are involved in the depolymerization of cellulose-hemicellulose framework^50^. Expansins (EXP), which lack significant hydrolase or transglycosylase activity^44^, are also thought to loosen cell wall hemicellulose–cellulose interactions^51^. *EXP1* is expressed at high levels specifically during tomato fruit ripening^52^. Antisense expression of *EXP1* resulted in firmer fruits, in contrast, fruits overexpressing high levels of *EXP1* were much softer than WT^44^. Minoia et al.^51^ demonstrated that a *SlEXP1* loss of function mutant yielded firmer and late ripening fruits through modification of hemicellulose structure. In tomato, XTH5 expression is highly related to fruit softening, although the biomechanical properties of plant walls could not be affected by incubation with SlXTH5^53^ and the function of SlXTH5 needs further verification. Previous discoveries have found that the CEL2 product contributed to cell wall disassembly occurring in cell separation during fruit abscission, but softening or textural changes in fruit pericarp was not affected by *CEL2* suppression^54^. *NOR-like1* knock-out resulted in firmer fruits, darker-colored and thicker, closely arranged microfilaments in *nor-like1* mutant cell walls, indicating that the cell wall relaxation and hemicelluloses-cellulose framework depolymerization were inhibited. NOR-like1 directly targeted *SlEXP1*, *SlCEL2* and activated their expression, which could explain this phenotype. Although the role of *CEL2* in tomato fruit softening is still controversial, the extremely low expression of *CEL2* in *nor-like1* mutants indicates its potential important role in cell wall modification.

Previous studies showed that *PG*^55,56^, *PME*^57,58^, and *EXP1*^52^ may be regulated by ethylene during tomato fruit ripening^29,37^, so the inhibition of pectin degradation and the xyloglucan-cellulose network depolymerization caused by the downregulated *PG* and *EXP1* expression in *nor-like1* mutants may be partly because of the reduction in ethylene synthesis (Fig. 8). Therefore, NOR-like1 affects fruit softening by regulating the dissolution of pectin, the depolymerization of cellulose-hemicellulose framework and the relaxation of cell wall and is a key regulator of tomato fruit softening.

## Materials and methods

### Plant materials and growth conditions

Wild-type tomato (*Solanum lycopersicum*) cultivars *Ailsa Craig* (AC) and transgenic lines were grown in the greenhouse. Flowers were tagged at anthesis to record the ripening stages accurately through fruit development. Fruits of wild-type and transgenic lines were collected at different ripening stages (MG, Br, B + 3, B + 6, and B + 9). Pericarp tissues of the fruits were collected after harvesting, frozen in liquid nitrogen immediately, and stored at −80℃ until use.

### Plasmid construction and VIGS assay

Tobacco rattle virus (TRV)-based vectors pTRV1 and pTRV2 were used for VIGS^59^. The *NOR-like1* cDNA fragment was amplified using the forward primer (5′-CTGCTTGGATCCCTCTCTCTTCTAAGCTGAAT-3′) and the reverse primer (5′-GACTTAGAATTCCAATATTCAAATCATCTCTT-3′), then inserted into the pTRV2 vector. The pTRV2-*NOR-like1* plasmid was verified by sequencing and transformed into GV3101. VIGS was carried out on AC fruits following the protocol as previously described^60^.

### Total RNA isolation and quantitative real-time PCR (qRT-PCR) analysis

Total RNA was isolated from the pericarp of tomato fruit according to the RNeasy Mini Kit (Qiagen, Germany) procedures and DNaseI (Qiagen, Germany) digestion was performed to remove genomic DNA. TransScript One-Step gDNA Removal and cDNA Synthesis SuperMix (TransGen, China) was used to synthesize cDNA from 2 μg total RNA. qRT-PCR was conducted using SYBR Green PCR Master Mix (TransGen, China) with a CFX96 Real-Time PCR System (Bio-Rad, USA). The tomato *Actin* gene (Solyc03g078400) was used as the internal control. Relative gene expression values were calculated according to the 2^−△△Ct^ method^61^. For each sample, three biological replicates were included. All primers used for qRT-PCR are listed in Supplemental Table S1.

### Subcellular localization analysis

The coding sequence (CDS) fragment of *NOR-like1* without the stop codon was amplified by PCR (primers used are listed in Supplemental Table S2) and then inserted into the pEAQ-GFP vector to produce the fusion construct pEAQ*-NOR-like1*-GFP using ClonExpress II One Step Cloning Kit (Vazyme, China). Then pEAQ-*NOR-like1*-GFP and the control vector (pEAQ-GFP) were transferred to *A. tumefaciens* strain GV3101 and injected into 4-week-old tobacco leaves^62^. GFP fluorescence was observed and captured by a laser confocal microscope (Leica, Germany) after 48 h of infiltration.

### pYLCRISPR/Cas9Pubi-H-*NOR-like1* vector construction and tomato genetic transformation

CRISPR-P (http://cbi.hzau.edu.cn/crispr/) was used to select four sgRNAs that targeted *NOR-like1* (Supplemental Table S3, Fig. 3a). The four sgRNAs were cloned into the pYLCRISPR/Cas9Pubi-H binary plasmid using the Golden Gate ligation method^63^. Oligonucleotide primers used in this section are listed in Supplemental Table S4. pYLCRISPR/Cas9Pubi-H-*NOR-like1* vector was transformed into AC using the stable *Agrobacterium tumefaciens*-mediated transformation method^64^. The transgenic tomato lines were selected through their hygromycin resistance.

### DNA extraction and mutation analysis

Total genomic DNA was extracted from tomato fresh frozen leaves using a DNA secure Plant Kit (Tiangen, China) and used as a template for amplifying the desired gene fragments using primers flanking the target sites. The PCR products were sequenced directly or cloned into the pEASY-T1 vector using pEASY-T1 Cloning Kit (TransGen, China) then sequenced to identify mutations. Oligonucleotide primers used for this analysis are listed in Supplemental Table S5 and Supplemental Table S6. For each target, two most likely off-target sites (Supplemental Table S7) were tested.

### Protein extraction and western blot

Proteins were extracted from tomato fruit pericarp as previously described with miner revisions^65^. Protein concentration was determined by BCA Protein Assay Kit (Solarbio, China). 30 μg of total proteins were separated by 12% SDS-PAGE and transferred to a PVDF membrane (Millipore, USA). The membrane was blocked in 5% nonfat milk in TBST buffer [20 mM Tris–HCl (pH 7.5), 150 mM NaCl and 0.1% Tween-20] at room temperature for 1 h. Immunoblots were performed at 4 °C overnight with affinity-purified rabbit polyclonal anti-NOR-like1 (the specific polyclonal antibodies against NOR-like1 were prepared with peptide antigen, and the peptide sequence is c-PIDHERDDLNIDMM, Abmart; 1:500 dilutions) or mouse monoclonal Anti-*β*-Actin (CWBIO, China; 1:2000 dilutions). The membranes were then washed by TBST buffer (3 × 10 min) and treated with the corresponding secondary antibodies (CWBIO, China; 1:10,000 dilutions) conjugated to horseradish peroxidase for 1 h at room temperature. Finally, the membranes were visualized using a horseradish peroxidase-enhanced chemiluminescence system (Solarbio, China).

### RNA sequencing and bioinformatics analysis

Total RNA was extracted from tomato fruits of WT and *nor-like1#1* mutant at B + 3 stage with the RNeasy Mini Kit (Qiagen, Germany), three biological replicates were made for each sample. The mRNA was enriched using oligo-dTs coupled with magnetic beads before being cut into 300 bp fragments (Novogene, China). Next, RNA-seq libraries were constructed and 150 bp pair-end sequencing was performed on HiSeq PE150 (Illumina, USA). The clean data were mapped to the tomato reference genome (version SL2.50) using TopHat software (version 2.0.14). Subsequently, fragments were assigned to genes by feature Counts and count programs, and gene expression abundance was represented by RPKM value. Differences in gene expression between *nor-like1* mutant and WT were identified by DESeq2 Library^66^. The fold change was calculated by RPKM_*slnac3*_/RPKM_WT_. Genes were considered as differentially expressed genes (DEGs) between NOR-like1 mutant and WT if∣fold change∣ ≥ 2 and *p*-value < 0.05.

### GO enrichment analysis

GO enrichment analysis was carried out by the GO seq R package^67^ based on DEGs, using a threshold of *p*-value < 0.05. Proteins were filtered based on their grouping to cellular components, molecular functions, and biological functions.

### Ethylene measurement

To measure ethylene production, fruit from WT and *nor-like1* mutants were harvested at different ripening stages (MG, Br, B + 3, B + 6, and B + 9), weighed, and placed at room temperature for 2 h to avoid measuring “wound ethylene” which was transiently generated by picking. Then the fruits were transferred into 300 ml gastight jars, sealed, and incubated at room temperature for 1 h. Then 1 ml gas samples were withdrawn and analyzed by a gas chromatograph equipped with a flame ionization detector. Ethylene concentrations were calculated by comparing sample peak areas with ethylene standards of known concentration, and normalized for fruit weight. At least three replicates were used for each measurement.

### Ethrel and 1-MCP treatment

The WT tomato fruits at the mature green stage were immersed in 0.4% ethrel or double distilled water (DDW) as control for 10 min^68^, then dried and placed at room temperature for 12 h. Wild-type fruits at breaker stage were treated with the ethylene signaling inhibitor 1-MCP (1.0 mg/l) or air as control for 16 h^69^. After treatment, the pericarp tissues were sliced, frozen in liquid nitrogen, and then used for RNA isolation and qRT-PCR. For each treatment, three biological replicates from independent sample were included.

### Carotenoids extraction and LC–Q-TOF–MS analysis

Carotenoids were extracted as described previously^70^ with minor revision. WT and *nor-like1* mutant fruits at B + 3 and B + 10 stages were used and four independent extractions were performed. Carotenoids were identified and the relative contents were determined as previously described^70^.

### Firmness measurement

The firmness of tomato fruits at five ripening stages (MG, Br, B + 3, B + 6, and B + 9) was measured by compressing a junction of outer and radial pericarp at the equator by using a 4 mm cylindrical probe (test speed 1 mm/s) (Brookfield CT3), avoiding visible vascular bundles, fissures and locular tissue. The maximum force developed during the test was recorded^71^. Each fruit was measured at two or three sites. The data for each fruit were averaged as one biological replicate and each measurement was performed using a minimum of three biological repetitions.

### Transmission electron microscope (TEM)

Pericarp samples were excised from WT and *nor-like1*#1 mutant fruits at the B + 3 stage, fixed and performed as previously described^72^. The sections were viewed in an FEI Talos 120C TEM (Hillsboro, OR) and micrographs were taken using the integrated Ceta CMOS camera.

### Electrophoretic mobility shift assay

The *NOR-like1* CDS region without the termination codon was inserted into the pGEX-GST vector by ClonExpress II One Step Cloning Kit (Vazyme, China), primers used are shown in Supplemental Table S2. Recombinant GST-tagged NOR-like1 protein was expressed in *E. coli* strain *Trans*etta DE3 (TransGen, China) and purified with Glutathione Sepharose 4B (GE Healthcare). EMSA was performed as previously described^73^ using the EMSA kit (Thermo Fisher Scientific, USA) according to the manufacturer’s instructions. The oligonucleotide probes containing the NACRS (NAC recognize sequence) [TA][TG][AGC]**CGT[GA]**[TA] and the mutant probes used in this study are listed in supplemental Table S8.

### Chromatin immunoprecipitation (ChIP)-qPCR analysis

Tomato fruits of WT and the *nor-like1#1* mutant at the breaker stage were harvested and cut into slices, immediately cross-linked with 1% (v/v) formaldehyde and ground into a fine powder in liquid nitrogen. The nuclei were isolated, and then sonicated to shear the DNA into 300–1000 bp fragments. Ten percent of total sonicated chromatin were reverse cross-linked and used as the input control. Polyclonal anti-NOR-like1 antibodies (Abmart, China) or equal amounts of IgG (Solarbio, China) were bound to Pierce™ ChIP-grade Protein A/G Magnetic Beads (Thermo Fisher Scientific, USA), and immunoprecipitated with corresponding sonicated chromatin overnight at 4 ℃ with rotation. Sonicated chromatin from WT fruits immunoprecipitated with IgG and sonicated chromatin from *nor-like1#1* mutant fruit immunoprecipitated with anti-NOR-like1 were both used as negative controls. After proteinase K treatment, crosslinking was reversed at 65 ℃ overnight in 0.25 M NaCl. Subsequently, the ChIP DNA was extracted, and the amount of each precipitated DNA fragment was determined by qRT-PCR using gene specific-primers listed in supplemental Table S9. The enrichment of predicted gene promoter fragments was normalized to its respective input DNA value. The error bars represent the SD from three independent experiments.

### Dual-luciferase reporter assay

Transcription activation of the *SlACS2*, *SlACS4*, *SlGgpps2*, *SlSGR1*, *SlPG2a*, *SlPL*, *SlCEL2*, and *SlEXP1* promoters by NOR-like1 was performed in tobacco (*N. benthamiana*) leaves using *Agrobacterium*-infiltration. The 1–2 kb promoter regions were cloned into pGreenII 0800-LUC double-reporter vector^74^, while the *NOR-like1* coding sequence was cloned into the pEAQ vector^75^ as effector. All primers used for vector construction are listed in supplemental Table S2. The recombinant vectors were sequenced and transferred into *A. tumefaciens* strain EHA105 separately. *A. tumefaciens* containing constructed effector and reporter plasmids were co-transformed into tobacco leaves and the LUC to REN ratio was calculated from the results of at least six transient assays.

### Statistical analysis

Significance analysis of corresponding experimental data was conducted using IBM SPSS statistics 20 software. Pairwise comparison was computed using Student’s *t*-test (*p < 0.05 and **p < 0.01), while multiple comparisons were subjected to ANOVA using Duncan test, statistically significant differences (*p* < 0.05) were indicated by diverse lowercase.

## Supplementary information

Supplemental Figures and Tables

Supplemental Data Set S1
