# Supplementary material for: A NAC transcription factor, NOR-like1, is a new positive regulator of tomato fruit ripening
Source: Hortic Res. 2018 Dec 21;5:75. doi: 10.1038/s41438-018-0111-5 (PMC6303401; doi:10.1038/s41438-018-0111-5)
Supplement: Supplementary file 1 — Supplemental Figures and Tables [file 41438_2018_111_MOESM1_ESM.docx]

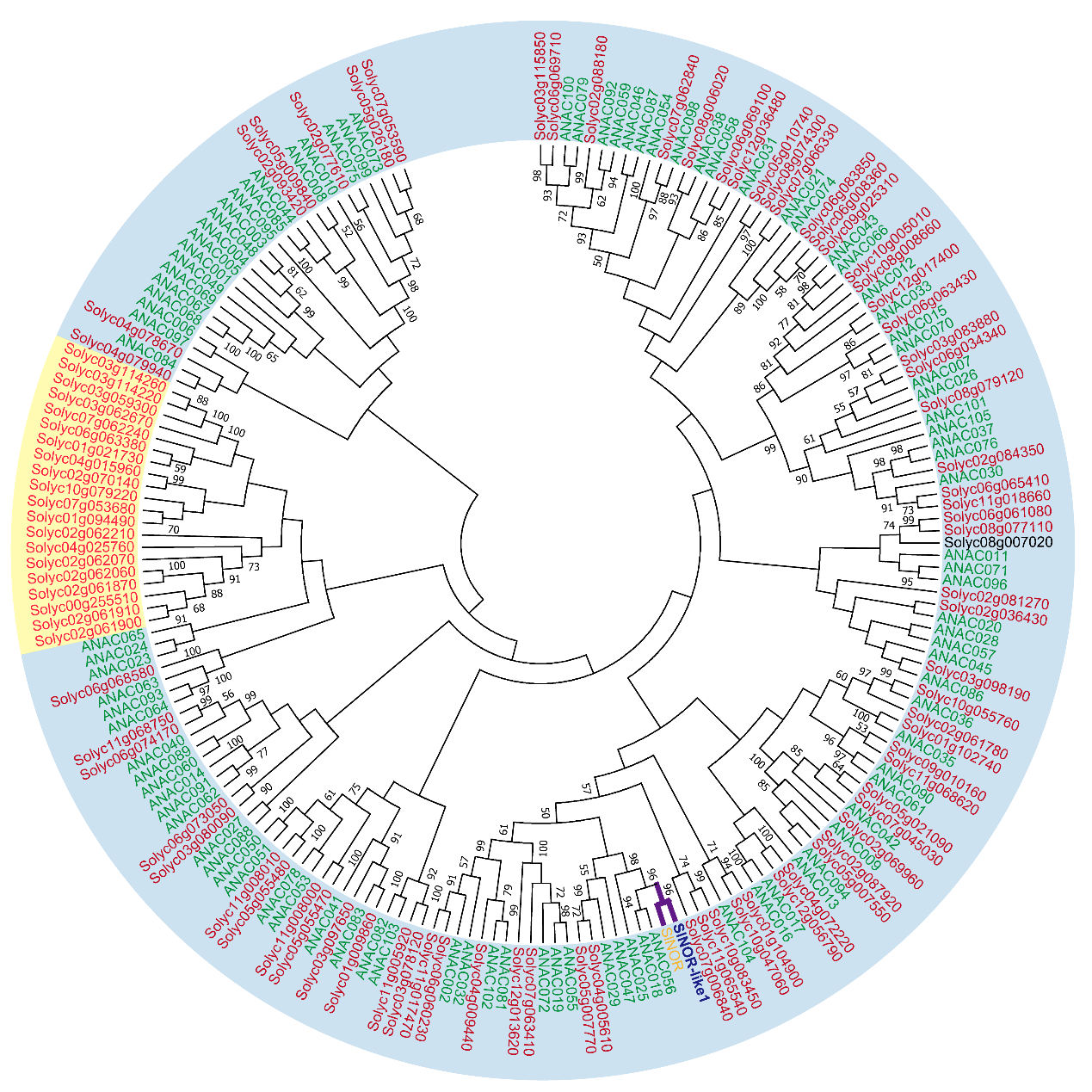


**Supplemental Figure S1. Evolutionary relationships of NAC family proteins from *Arabidopsis* and tomato.**

The evolutionary history was inferred using the Neighbor-Joining method. The percentage of replicate trees in which the associated taxa clustered together in the bootstrap test (1000 replicates) is shown next to the branches. The tree is drawn to scale, with branch lengths in the same units as those of the evolutionary distances used to infer the phylogenetic tree. The evolutionary distances were computed using the Poisson correction method and are in the units of the number of amino acid substitutions per site. Evolutionary analyses were conducted in MEGA7. Yellow indicates the tNAC subgroup which is unique to tomato.


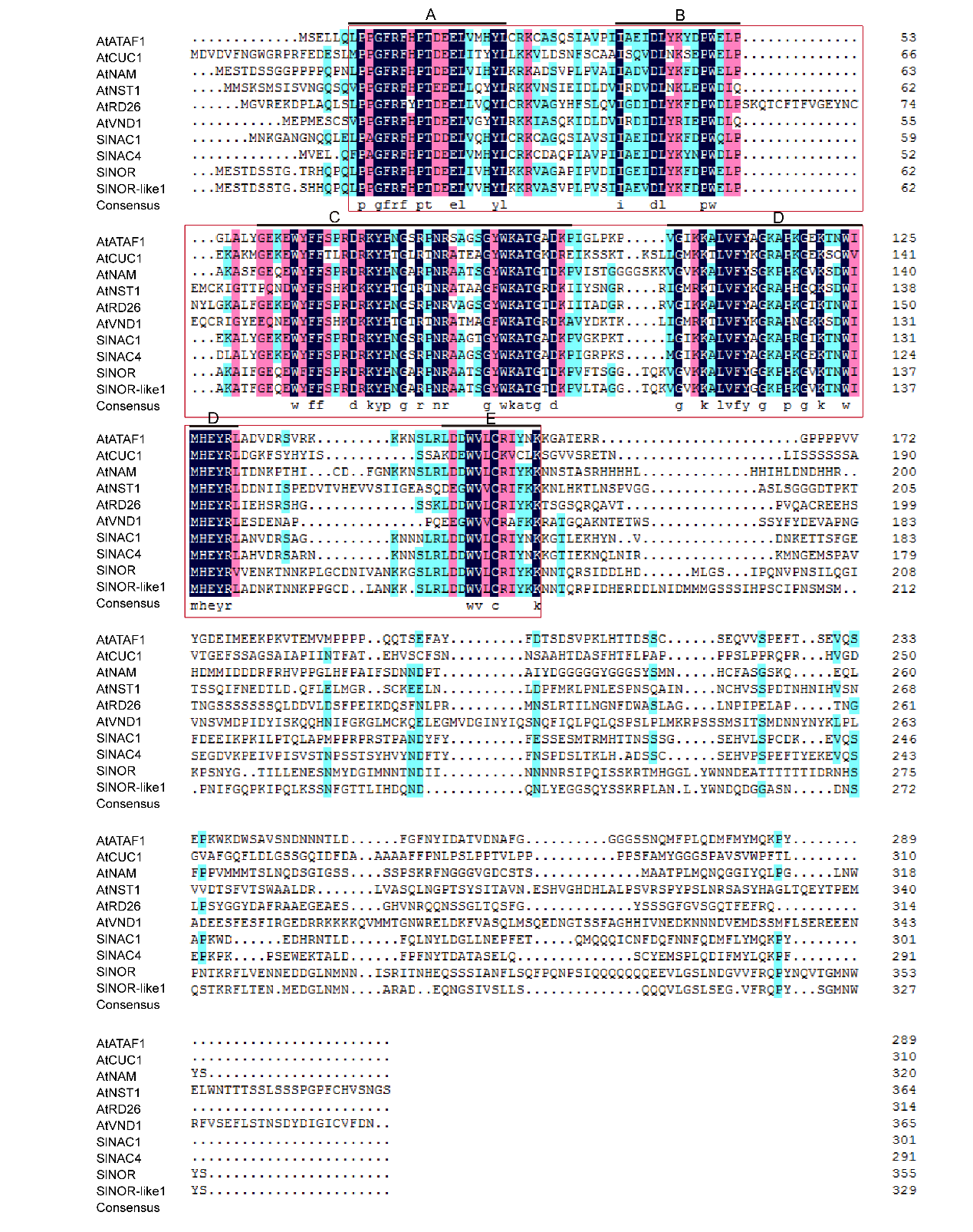


**Supplemental Figure S2. Multiple sequence alignment of NOR-like1 and other known NAC domain proteins from *Arabidopsis* and tomato.**

The alignment result was displayed using DNAMAN software. The red rectangular box indicates the NAC domain. The locations of the five highly conserved subdomains (A–E) are indicated with black lines on top of the sequences. At, *Arabidopsis thaliana*, Sl, *Solanum lycopersicum*.


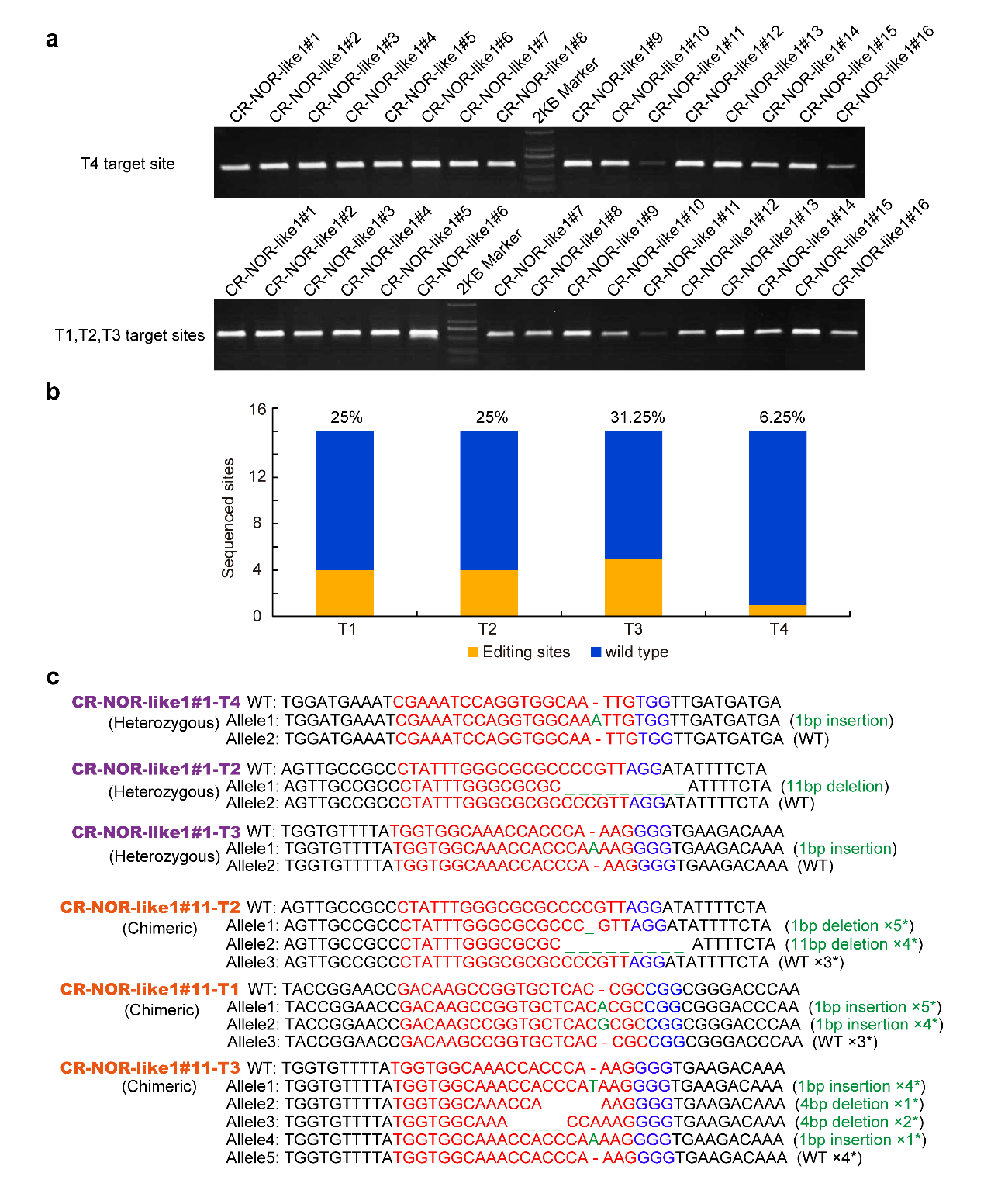


**Supplemental Figure S3. Gene editing analysis of T0 generation.**

**a** Agarose electrophoresis analysis of T4, T1, T2 and T3 target fragments amplified using PCR. **b** Editing rate of four target sites. **c** Gene editing analysis of CR-NOR-like1#1 and CR-NOR-like1#11. Red letters indicate the target sites, green letters represent edited site and editing type, and blue letters represent the protospacer adjacent motif (PAM).


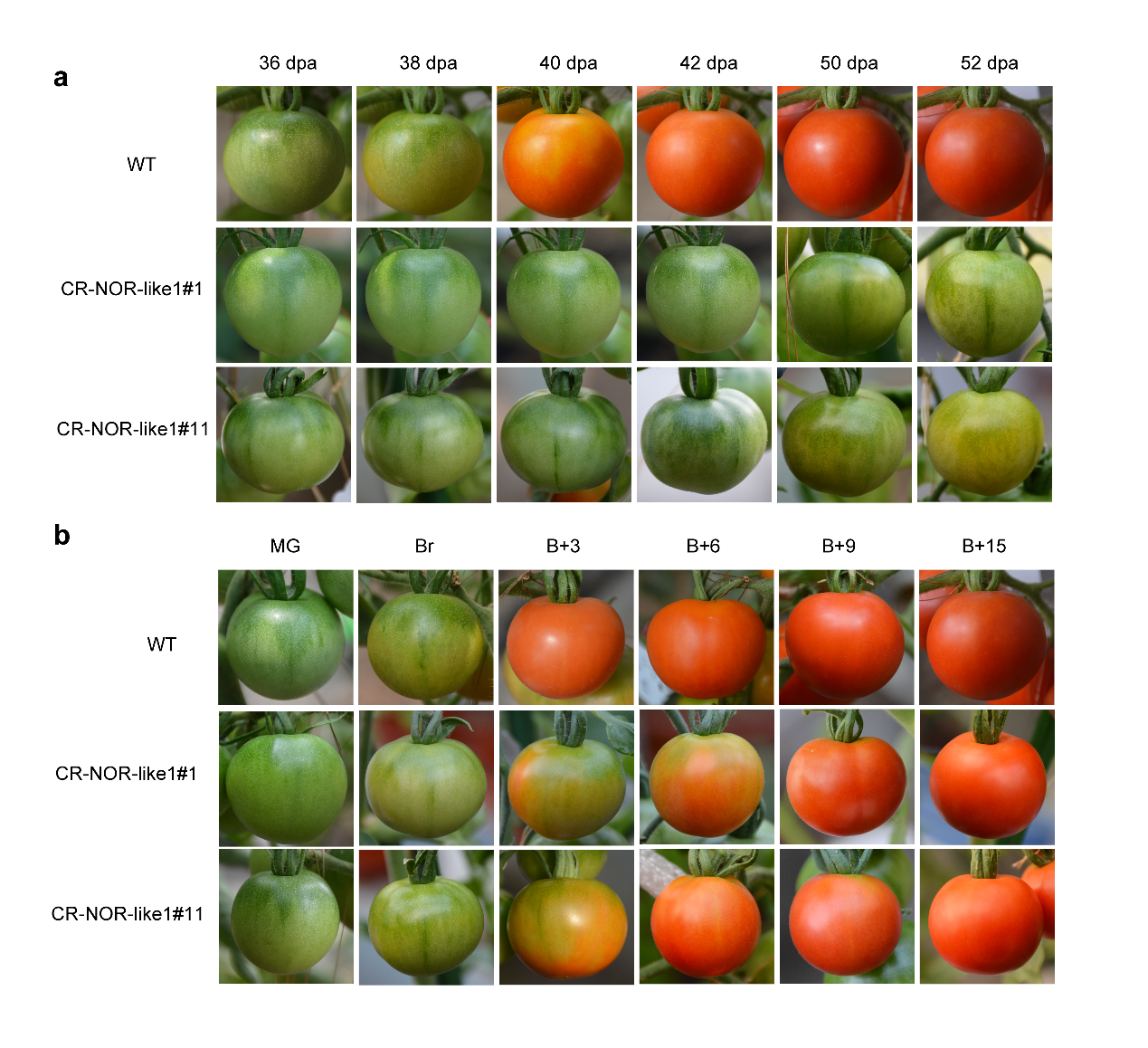


**Supplemental Figure S4. Loss-of-function of NOR-like1 significantly delayed tomato fruit ripening in T0 generation.**

**a** The time from anthesis to the breaker stage was delayed 14 days (CR-NOR-like1#1) and 12 days (CR-NOR-like1#11) compared to that of the wild type. dpa: days post anthesis. **b** The ripening process after breaker was inhibited in both CR-NOR-like1#1 and CR-NOR-like1#11 transgenic lines. MG: mature green; Br: breaker; B+3: 3 days after breaker; B+6: 6 days after breaker; B+9: 9 days after breaker; B+15: 15 days after breaker.


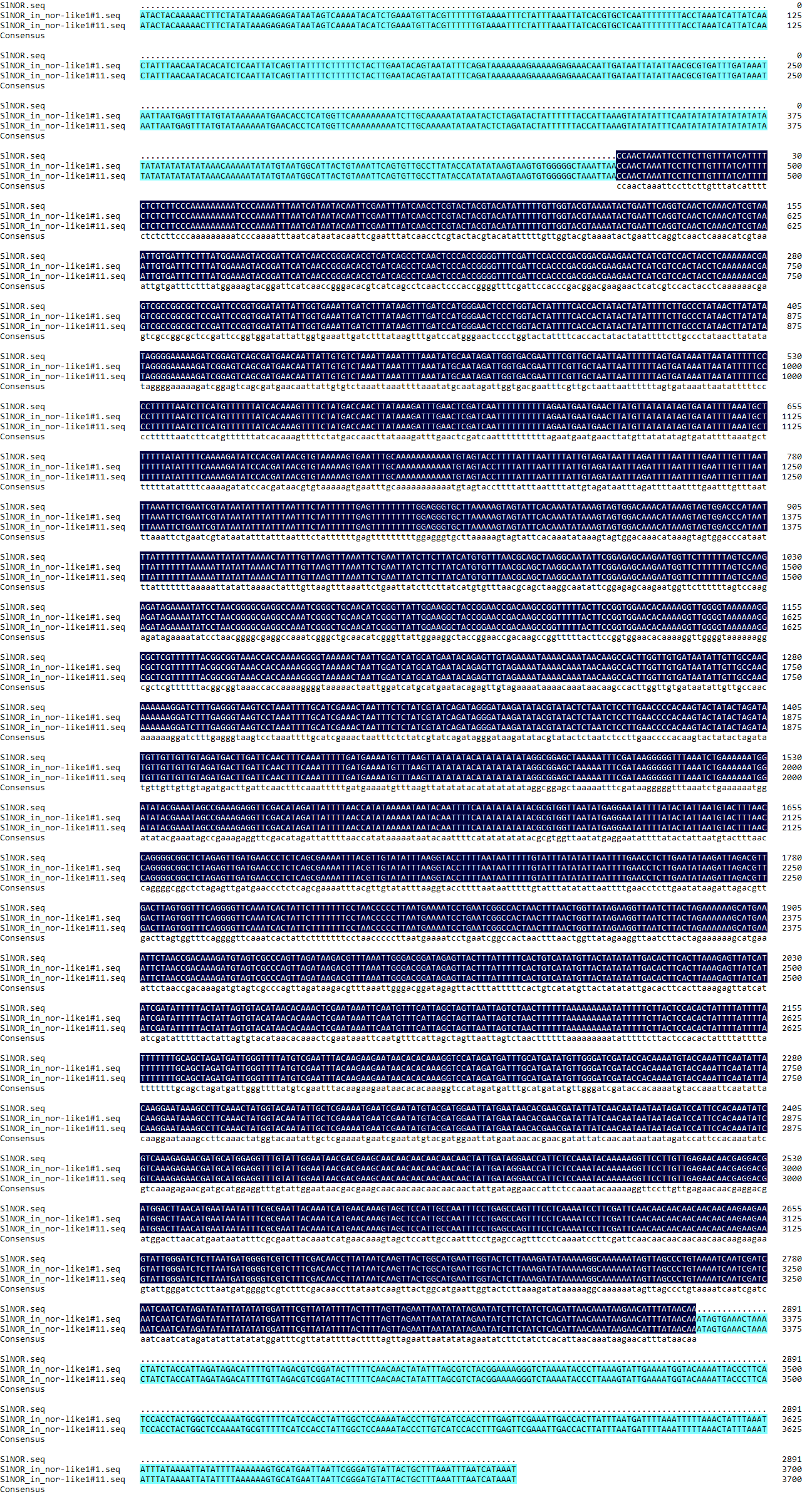


**Supplemental Figure S5.** **Sequencing of *Nor* gene in *nor-like1*#1 and *nor-like1*#11 mutants.**


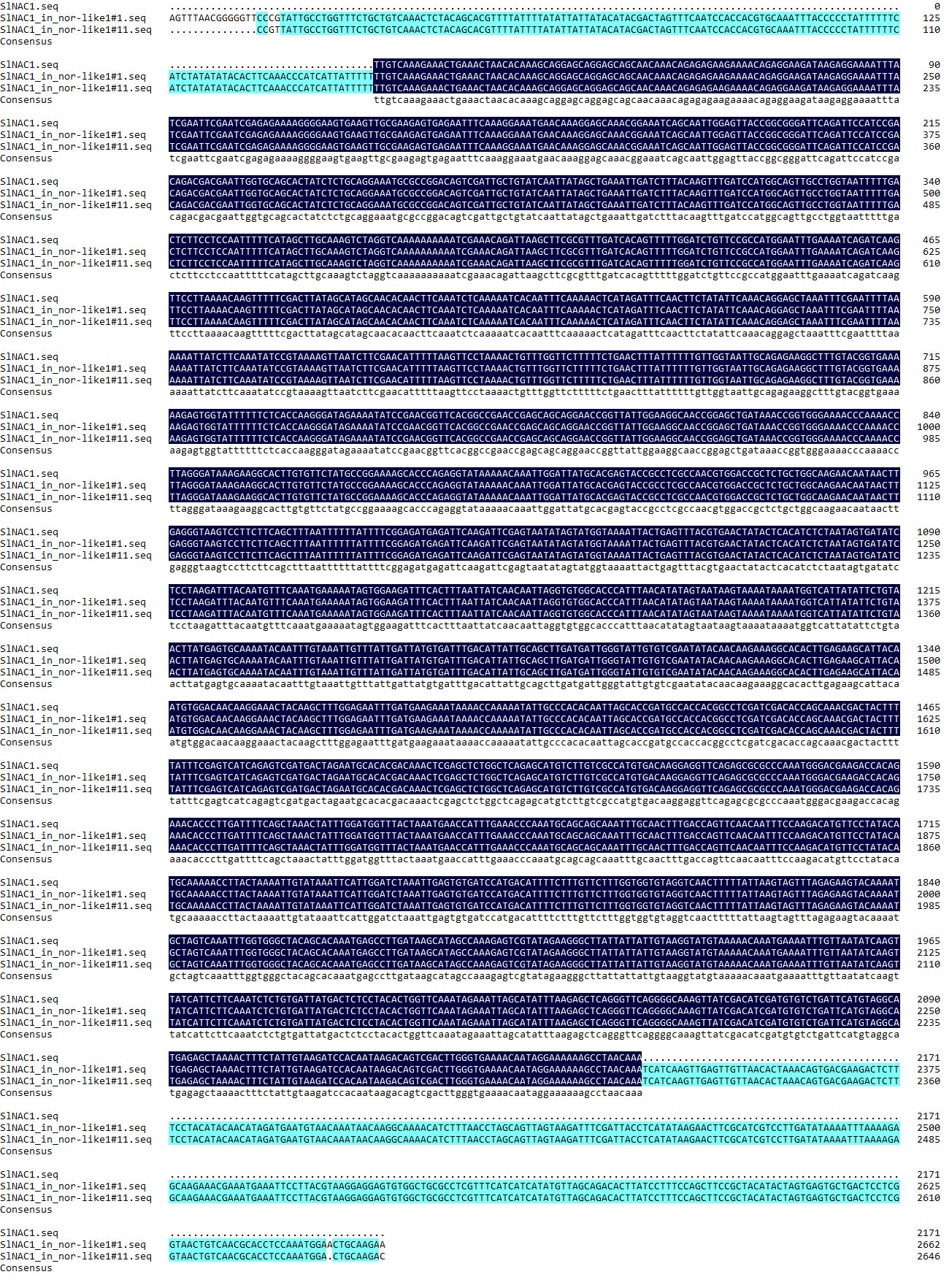


**Supplemental Figure S6. Sequencing of *SlNAC1* gene in *nor-like1*#1 and *nor-like1*#11 mutants.**


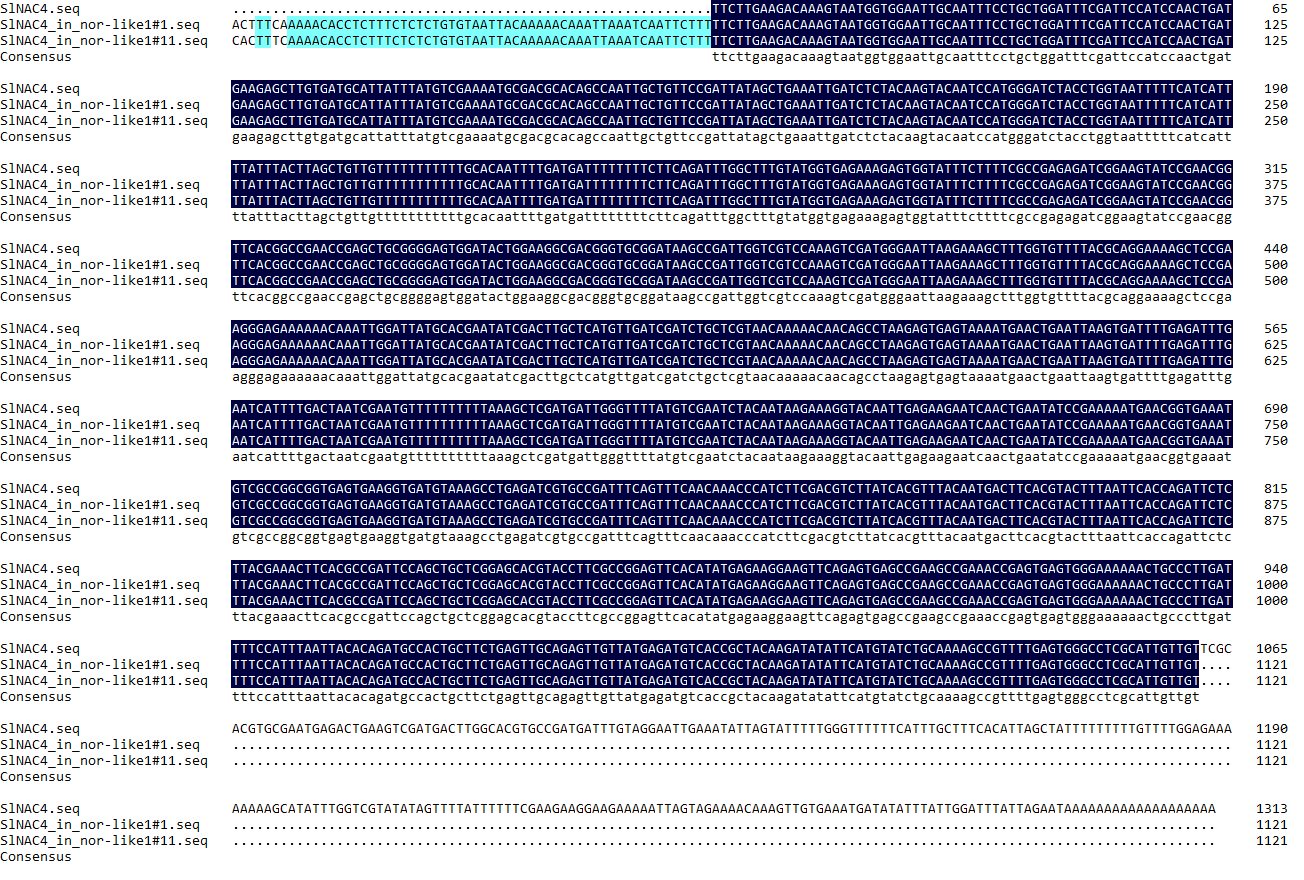


**Supplemental Figure S7. Sequencing of *SlNAC4* gene in *nor-like1*#1 and *nor-like1*#11 mutants.**


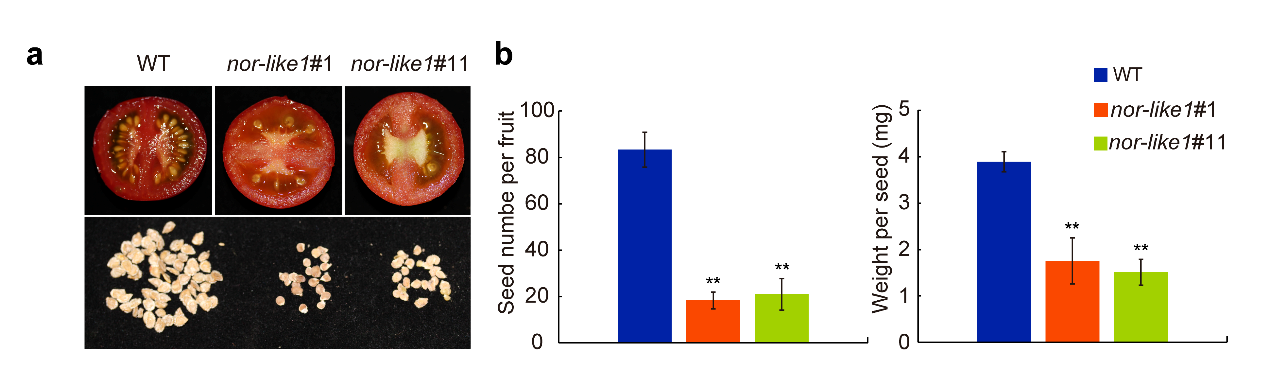


**Supplemental Figure S8. Seed development was abnormal in *nor-like1* mutants.**

**a** Both *nor-like1*#1 and *nor-like1*#11 mutants exhibited abnormal seed morphology. **b** The average seed number in *nor-like1*#1 and *nor-like1*#11 mutants was less than WT, and the seed weight was lighter in *nor-like1*#1 and *nor-like1*#11 mutants compared with WT. Bars represent ± SD of six independent replicates. ** indicate P<0.01 (Student’s t-test).


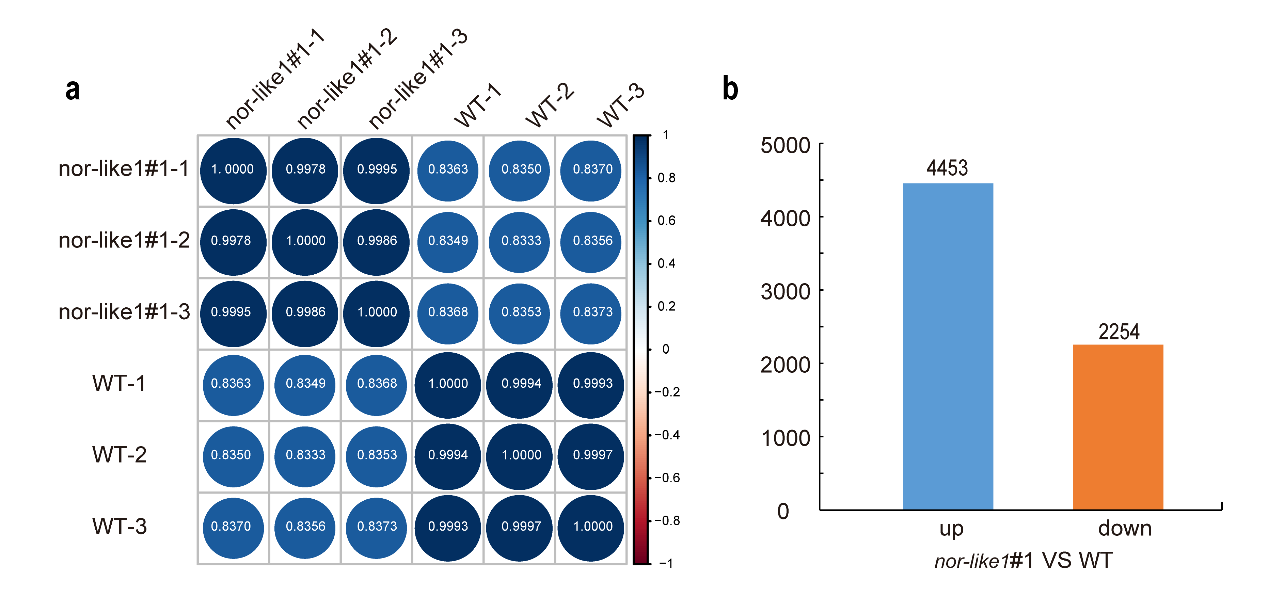


**Supplemental Figure S9. The quality of RNA-seq data was high and many down-regulated and up-regulated genes were found.**

**a** The correlation analysis of RNA-seq data between *nor-like1*#1 and WT. The plot color changes from red to blue (-1, 1) represents the correlation from low to high. **b** The number of genes which were up-regulated and down-regulated in *nor-like1*#1 compared with WT.


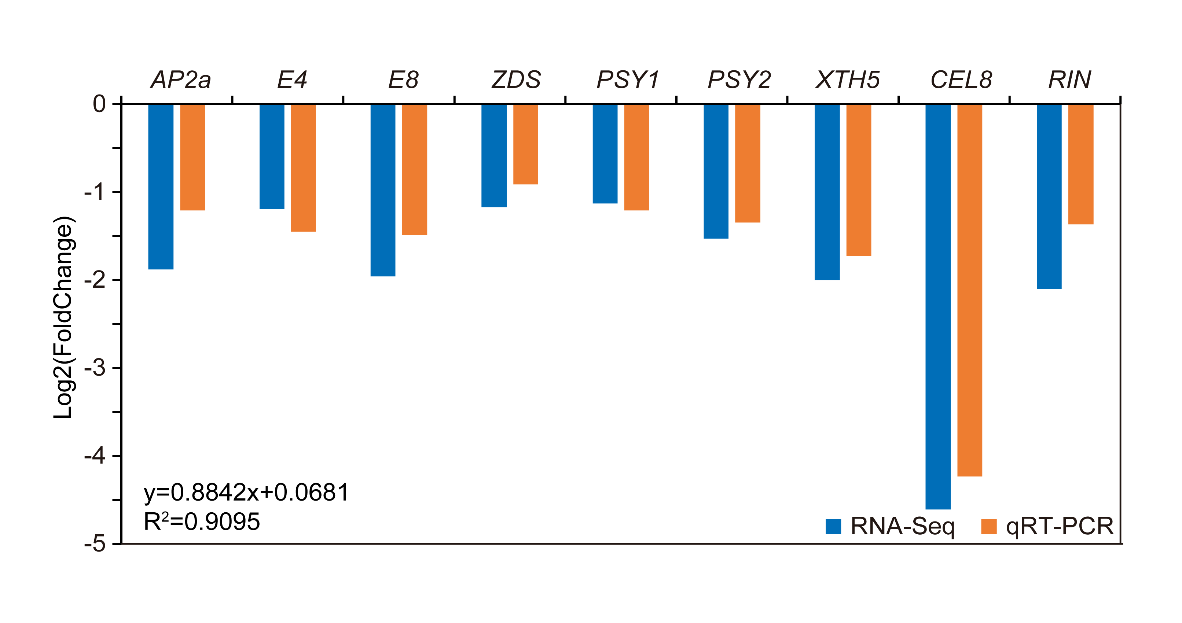


**Supplemental Figure S10. The correlation coefficient between RNA-seq and qRT-PCR.**

The Log2 (Fold Change) of 9 genes expression in *nor-like1*#1 and WT with the RNA-seq and qRT-PCR methods. RNA-seq data is indicated in the blue column and qRT-PCR data is indicated in the orange column. The Pearson correlation R^2^ is 0.9095 between RNA-seq data and qRT-PCR data.

**Supplemental Table S1. Primers used for qRT-PCR.**

| **Gene** | **Primer** | **Sequence (5’-3’)** |
| --- | --- | --- |
| *NOR-like1* | For | CCAACTGATGAAGAGCTTGTGG |
|  | Rev | AGCAGGTAGTTCCCAAGGATCA |
| *SlACS2* | For | AAACCCCAACGGAGTTATCC |
|  | Rev | GCAATGGCCTTGAATGATTT |
| *SlACS4* | For | TGGATTGCCTGAATTCACAA |
|  | Rev | ATCACCAGGATCAGCCAAAC |
| *SlAP2a* | For | AGAAATGGGGGACAATAGGG |
|  | Rev | ATTGTTGCTGCTCGGAGTCT |
| *SlE4* | For | TCTAAATCGCCAGGGTAATGAT |
|  | Rev | TAGCTTCTAACGACTCCCTTGC |
| *SlE8* | For | TTACATGGCTCCGAATCCTC |
|  | Rev | CGAGACCGAGACCTTCAGAC |
| *SlGgpps2* | For | GGGATTGGAAAAGGCTAAGG |
|  | Rev | AGCAATCAATGGAGCAGCTT |
| *SlZDS* | For | TGCTTCAATGTGTCCTTACACC |
|  | Rev | TGACCAGGTAACCTCAAGACCT |
| *SlPSY1* | For | GCTAATGACTCCCGAGAGAAGA |
|  | Rev | CGGGGTAATATATGATGCGTTT |
| *SlPSY2* | For | GGAGAAGATGCAAGAAGAGGAA |
|  | Rev | ATCAGTCACCTTTCCAGCAAAT |
| *SlSGR1* | For | TGGCTATCTCCCAAACCATC |
|  | Rev | GTGGCAATGGACATGAAGTG |
| *SlPG2a* | For | AGCTAAGGGTGATGGAAAAACA |
|  | Rev | TGAAAAGGTGATTTGCTTGAGA |
| *SlPL* | For | ATTGGTGGAAGTGCTGATCC |
|  | Rev | GTGCATCCTCGTGCTTTGTA |
| *SlCEL2* | For | AAACACATTGCCAAACGTCA |
|  | Rev | CCACAGATGGCACAGAGCTA |
| *SlEXP1* | For | AAATGACAATGGTGGCTGGT |
|  | Rev | CTGATTCCTCCTTGCTTTCG |
| *SlXTH5* | For | ATTCAGCCATCTCTTTGGTGAT |
|  | Rev | ACTTGAACCCTGAACCTGTGTT |
| *SlCEL8* | For | GCAAGACTTCATGCTGGTCA |
|  | Rev | GCATTAGCTGGGACTGGAAG |
| *SlRIN* | For | AACATCATGGCATTGTGGTG |
|  | Rev | TGTTGATGGTGCTGCATTTT |

**Supplemental Table S2. Primers used for vector construction.**

| **Assay** | **Primer** | **Sequence (5’-3’)** |
| --- | --- | --- |
| Subcellular Localization | pEAQ-NOR-like1-GFP-F | ctgcccaaattcgcgaccggtATGGAGAGTACCGATTCATCAACC |
|  | pEAQ-NOR-like1-GFP-R | tcctttgctagtcataccggtAGAGTACCAATTCATGCCTGAATAAG |
| DLR | pEAQ-NOR-like1-F | ctgcccaaattcgcgaccggtATGGAGAGTACCGATTCATCAACC |
|  | pEAQ-NOR-like1-R | accagagttaaaggcctcgagTTAAGAGTACCAATTCATGCCTGAA |
|  | 0800-*Sl*ACS2-F | ctatagggcgaattgggtaccATTACCAGTAGTACCATTGTATTCTCAACT |
|  | 0800-*Sl*ACS2-R | tgtttttggcgtcttccatggTGTGAGGGATATATATAAGGGGAAAG |
|  | 0800-*Sl*ACS4-F | ctatagggcgaattgggtaccCAGTTAATAATTCAAAATATTTAAAAAATATTCG |
|  | 0800-*Sl*ACS4-R | tgtttttggcgtcttccatggGAATGAAGAAGCTATGAGGGGTTT |
|  | 0800-*Sl*SGR1-F | ctatagggcgaattgggtaccAAAATTGTTTCTTTCTTAATTAGGGTATCA |
|  | 0800-*Sl*SGR1-R | tgtttttggcgtcttccatggTTTTTCTACAAGAAATTGGGTACCC |
|  | 0800-*Sl*Ggpps2-F | ctatagggcgaattgggtaccTCACCTTTATATTTTTTAATTTTGGGTT |
|  | 0800-*Sl*Ggpps2-R | tgtttttggcgtcttccatggTTTCTTTTCCAAAAAGCTAAGCTTT |
|  | 0800-*Sl*PG2a-F | ctatagggcgaattgggtaccTTTAAAAAAAATTTGGGGCTTTG |
|  | 0800- *Sl*PG2a -R | tgtttttggcgtcttccatggGATATATTGTTATATGGTATGGTTTTTAAACTT |
|  | 0800-*Sl*PL-F | ctatagggcgaattgggtaccTTGGGGTTGATAGAAATAGGGAAG |
|  | 0800-*Sl*PL-R | tgtttttggcgtcttccatggTATATATACATCGTTCAATATCATTACGTAAAA |
|  | 0800-*Sl*EXP1-F | ctatagggcgaattgggtaccTAAAAGGGGGCCACATCCA |
|  | 0800-*Sl*EXP1-R | tgtttttggcgtcttccatggCAAAAACAAAATTCAAAACTCAAATATAA |
|  | 0800-*Sl*CEL2-F | ctatagggcgaattgggtaccCATGCAAATGATAGGGCCAAT |
|  | 0800-*Sl*CEL2-R | tgtttttggcgtcttccatggTATTTGGTTGTGAACTGAGGGAAA |
| EMSA | pGEX-NOR-like1-F | ccgcgtggatccccggaattcATGGAGAGTACCGATTCATCAACC |
|  | pGEX-NOR-like1-R | gtcacgatgcggccgctcgagTTAAGAGTACCAATTCATGCCTGAA |

**Supplemental Table S3. Sequence of target sites.**

| **Gene** | **sequence (5’-3’)** |
| --- | --- |
| *NOR-like1* | T1：GACAAGCCGGTGCTCACCGC |
|  | T2：CTATTTGGGCGCGCCCCGTT |
|  | T3：TGGTGGCAAACCACCCAAAG |
|  | T4：CGAAATCCAGGTGGCAATTG |

**Supplemental Table S4. Primers used for recombinant pYLCRISPR/Cas9Pubi-H-*NOR-like1* vector construction.**

| **PCR** | **Primer** | **Sequence (5’-3’)** |
| --- | --- | --- |
| 1^st^ PCR | U-F | CTCCGTTTTACCTGTGGAATCG |
|  | gR-R | CGGAGGAAAATTCCATCCAC |
|  | AtU3d-NOR-like1 T1 Rev | GCGGTGAGCACCGGCTTGTCTGACCAATGGTGCTTTG |
|  | gR-NOR-like1 T1 For | GACAAGCCGGTGCTCACCGCGTTTTAGAGCTAGAAAT |
|  | AtU3b-NOR-like1 T2 Rev | AACGGGGCGCGCCCAAATAGTGACCAATGTTGCTCC |
|  | gR-NOR-like1 T2 For | CTATTTGGGCGCGCCCCGTTGTTTTAGAGCTAGAAAT |
|  | AtU6-1 SlAC3 T3 Rev | CTTTGGGTGGTTTGCCACCACaatcactacttcgtct |
|  | gR-NOR-like1 T3 For | TGGTGGCAAACCACCCAAAGgttttagagctagaaat |
|  | AtU6-29 NOR-like1 T4 Rev | CAATTGCCACCTGGATTTCGCaatctcttagtcgact |
|  | gR-NOR-like1 T4 For | CGAAATCCAGGTGGCAATTGgttttagagctagaaat |
| 2^nd^ PCR | Pps-GGL | TTCAGAggtctcTctcgACTAGTATGGAATCGGCAGCAAAGG |
|  | Pgs-GG2 | AGCGTGggtctcGtcagggTCCATCCACTCCAAGCTC |
|  | Pps-GG2 | TTCAGAggtctcTctgacacTGGAATCGGCAGCAAAGG |
|  | Pgs-GG3 | AGCGTGggtctcGtcttcacTCCATCCACTCCAAGCTC |
|  | Pps-GG3 | TTCAGAggtctcTaagacttTGGAATCGGCAGCAAAGG |
|  | Pgs-GG4 | AGCGTGggtctcGagtccttTCCATCCACTCCAAGCTC |
|  | Pps-GG4 | TTCAGAggtctcTgactacaTGGAATCGGCAGCAAAGG |
|  | Pgs-GGR | AGCGTGggtctcGaccgACGCGTATCCATCCACTCCAAGCT |

**Supplemental Table S5. Primers used for target site mutation analysis.**

| **Target site** | **Primer** | **Sequence (5’-3’)** |
| --- | --- | --- |
| NOR-like1-T2,T1,T3 | For | CCTTTCCTTCTTCTCTCACGTCA |
|  | Rev | TATTCCAACAAATGGTGTGCTGT |
|  | Seq1 | CCTTTCCTTCTTCTCTCACGTCA |
|  | Seq2 | TATTCCAACAAATGGTGTGCTGT |
| NOR-like1-T4 | For | AACCAAACCAATTTCCAGCTG |
|  | Rev | CCACGAAAGATTAAAGTTCCTGTG |
|  | Seq | TACATACCAGGTAGTTCCCAAG |

**Supplemental Table S6. Primers used for off-target site mutation analysis.**

| **Off-target site** | **Primer** | **Sequence(5’-3’)** |
| --- | --- | --- |
| NOR-like1-T2  off-target site 1 | For | GACAAACATAAAGTAGTGGACC |
|  | Rev | GGGTTCAAGGAGATTAGAGTAT |
|  | Seq | GTGTTTAACGCAGCTAAGGC |
| NOR-like1-T3  off-target site 1 | For | TTCTCATCCAGTAAATAGCCGTAC |
|  | Rev | ACCTCCTTCGCAATCACC |
|  | Seq | GCCTTTGTCAACATCCTCATT |
| NOR-like1-T3  off-target site 2 | For | GACAAACATAAAGTAGTGGACC |
|  | Rev | GGGTTCAAGGAGATTAGAGTAT |
|  | Seq | GTGTTTAACGCAGCTAAGGC |
| NOR-like1-T4  off-target site 1 | For | CTTTCAACTGGCGGAGGA |
|  | Rev | CTGCCATTGAACGCCCTA |
|  | Seq | GAGGGTGGGCTCTTTAGG |
| NOR-like1-T4  off-target site 2 | For | TGCTAGTTTTCTTCGTCTCTT |
|  | Rev | GTCCACGAGACAGGAATAGA |
|  | Seq | TAGTAAGCCGTTTAGGAAGACA |

**Supplemental Table S7. Detection of mutations on putative off-target sites.**

| **Target** | **Name of putative off-target site** | **Putative off-target locus** | **Sequence of the putative off-target site** | **No. of T1 transgenic lines sequenced** | **No. of plants with mutations** |
| --- | --- | --- | --- | --- | --- |
| *NOR-like1*  *-*T2 | OFF1 | SL2.50ch10: +1307462 | CGATTTGGCCTCGCCCCGTTAGG | 2 | 0 |
| *NOR-like1*  *-*T3 | OFF1 | SL2.50ch03:+63250064 | GTGTTGCAAAGCACCCAAAGAGG | 2 | 0 |
|  | OFF2 | SL2.50ch10: -1307326 | CGGCGGTAAACCACCAAAAGGGG | 2 | 0 |
| *NOR-like1*  -T4 | OFF1 | SL2.50ch01:-92700991 | CGAAGTCCAGGTGGCAAATCCAG | 2 | 0 |
|  | OFF2 | SL2.50ch01:+86994085 | TGGAATCCAGGGGGCAATTACGG | 2 | 0 |

a, mismatching bases are marked in red.

**Supplemental Table S8. Probes containing NACRS used in EMSA.**

| **Gene** | **Primer** | **Sequence(5’-3’)** |
| --- | --- | --- |
| *SlACS2* | wild-F | AATAAATTCTTTTTGACAGGGTGGCGTAAAAATAATTTTATTTTAAAATA |
|  | wild-R | TATTTTAAAATAAAATTATTTTTACGCCACCCTGTCAAAAAGAATTTATT |
|  | mutant-F | AATAAATTCTTTTTGACAGGGAAAAAAAAAAATAATTTTATTTTAAAATA |
|  | mutant-R | TATTTTAAAATAAAATTATTTTTTTTTTTCCCTGTCAAAAAGAATTTATT |
| *SlACS4* | wild-F | GTTTTAACTTTTTAAATATTTTGACGTAAGTTTTTTGTTTTCCCCCAAAA |
|  | wild-R | TTTTGGGGGAAAACAAAAAACTTACGTCAAAATATTTAAAAAGTTAAAAC |
|  | mutant-F | GTTTTAACTTTTTAAATATTTAAAAAAAAGTTTTTTGTTTTCCCCCAAAA |
|  | mutant-R | TTTTGGGGGAAAACAAAAAACTTTTTTTTAAATATTTAAAAAGTTAAAAC |
| *SlSGR1* | wild-F | TAAGCAGTTATGCGTGTGCTGAAGTTGTTATTTCAAACGTGAAAAGGAAA |
|  | wild-R | TTTCCTTTTCACGTTTGAAATAACAACTTCAGCACACGCATAACTGCTTA |
|  | mutant-F | TAAGCAGTTAAAAAAAAGCTGAAGTTGTTATTTCAAAAAAAAAAAGGAAA |
|  | mutant-R | TTTCCTTTTTTTTTTTGAAATAACAACTTCAGCTTTTTTTTAACTGCTTA |
| *SlGgpps2* | wild-F | TACGTTTAGGATAGGACTTCATTACGTGTCAAGGACAAAAATAAACCTAT |
|  | wild-R | ATAGGTTTATTTTTGTCCTTGACACGTAATGAAGTCCTATCCTAAACGTA |
|  | mutant-F | TACGTTTAGGATAGGACTTCAAAAAAAAACAAGGACAAAAATAAACCTAT |
|  | mutant-R | ATAGGTTTATTTTTGTCCTTGTTTTTTTTTGAAGTCCTATCCTAAACGTA |
| *SlPG2a* | wild-F | TATTTTAAAAAATACACTTAAAAGCGTGACTAAATATAAATTGTTGCTCA |
|  | wild-R | TGAGCAACAATTTATATTTAGTCACGCTTTTAAGTGTATTTTTTAAAATA |
|  | mutant-F | TATTTTAAAAAATACACTTAAAAAAAAAACTAAATATAAATTGTTGCTCA |
|  | mutant-R | TGAGCAACAATTTATATTTAGTTTTTTTTTTAAGTGTATTTTTTAAAATA |
| *SlPL* | wild-F | TATCAATAACAGATAGTAATTTTACGTAATGATATTGAACGATGTATATA |
|  | wild-R | TATATACATCGTTCAATATCATTACGTAAAATTACTATCTGTTATTGATA |
|  | mutant-F | TATCAATAACAGATAGTAATTAAAAAAAATGATATTGAACGATGTATATA |
|  | mutant-R | TATATACATCGTTCAATATCATTTTTTTTAATTACTATCTGTTATTGATA |
| *SlCEL2* | wild-F | ATCCAATAGCATCCCACCACGTGTCGTAACAATATTGGTTTAACCCCAGG |
|  | wild-R | CCTGGGGTTAAACCAATATTGTTACGACACGTGGTGGGATGCTATTGGAT |
|  | mutant-F | ATCCAATAGCATCCCACCAAAAAAAAAAACAATATTGGTTTAACCCCAGG |
|  | mutant-R | CCTGGGGTTAAACCAATATTGTTTTTTTTTTTGGTGGGATGCTATTGGAT |
| *SlEXP1* | wild-F | CTGAAATTTTAGCTAATTTTAACACGTATTCAATTTTGTATGATTGAATG |
|  | wild-R | CATTCAATCATACAAAATTGAATACGTGTTAAAATTAGCTAAAATTTCAG |
|  | mutant-F | CTGAAATTTTAGCTAATTTTAAAAAAAAATCAATTTTGTATGATTGAATG |
|  | mutant-R | CATTCAATCATACAAAATTGATTTTTTTTTAAAATTAGCTAAAATTTCAG |

**Supplemental Table S9. Primers used for ChIP-qPCR.**

| **Gene** | **Primer** | **Sequence(5’-3’)** |
| --- | --- | --- |
| *SlACS2* | For | TTTTGACAGGGTGGCGTA |
|  | Rev | CCCTAGCCTCGAACCAAC |
| *SlACS4* | For | TTTCCCCCAAAAGTTTCACA |
|  | Rev | CCGAGATTAAAACATGGCAGA |
| *SlGgpps2* | For | GGACTTCATTACGTGTCAAGGAC |
|  | Rev | TTTAACTTTGCAATCTATTGACTTTT |
| *SlSGR1* | For | AAAATAAGCAGTTATGCGTGTG |
|  | Rev | AACCCCTAACATTTCTTTCTCC |
| *SlCEL2* | For | CCGGTCCATCAAAAGTAGGA |
|  | Rev | TCATTGGATTCCTGGGGTTA |
| *SlPL* | For | CGTATACACGTTTAATTGACAT |
|  | Rev | GATTACTACTCTAGCATACGTCCA |
| *SlPG2a* | For | TTTAGTCACGCTTTTAAGTGTATTTTT |
|  | Rev | CGTTTGATATTTTTGGCAATCA |
| *SlEXP1* | For | TGGAAAAATTGAAAGGGTGTT |
|  | Rev | GAATTTTCCGCTCCCACTATT |
